# Recent levels and trends in HIV incidence rates among adolescent girls and young women in ten high-prevalence African countries: a systematic review and meta-analysis

**DOI:** 10.1016/S2214-109X(19)30410-3

**Published:** 2019-10-10

**Authors:** Isolde Birdthistle, Clare Tanton, Andrew Tomita, Kristen de Graaf, Susan B Schaffnit, Frank Tanser, Emma Slaymaker

**Affiliations:** a Department of Population Health, London School of Hygiene & Tropical Medicine, London, UK; b Department of Infectious Disease Epidemiology, London School of Hygiene & Tropical Medicine, London, UK; c Centre for Rural Health, School of Nursing and Public Health, and KwaZulu-Natal Research Innovation and Sequencing Platform (KRISP), College of Health Sciences, University of KwaZulu-Natal, Durban, South Africa; d Africa Health Research Institute, Africa Centre Building, Mtubatuba, South Africa

## Abstract

**Background:**

The roll-out of antiretroviral therapy (ART) has changed contexts of HIV risk, but the influence on HIV incidence among young women is not clear. We aimed to summarise direct estimates of HIV incidence among adolescent girls and young women since ART and before large investments in targeted prevention for those in sub-Saharan Africa.

**Methods:**

We did a systematic review and meta-analysis. We searched MEDLINE, Embase, Web of Science, Global Health, and CINAHL for studies reporting HIV incidence data from serological samples collected among females aged 15–24 years in ten countries (Kenya, Lesotho, Malawi, Mozambique, South Africa, Swaziland, Tanzania, Uganda, Zambia, and Zimbabwe) that were selected for DREAMS investment in 2015. We only included articles published in English. Our main outcome was to summarise recent levels and trends in HIV incidence estimates collected between 2005 and 2015, published or received from study authors, by age and sex, and pooled by region.

**Findings:**

51 studies were identified from nine of the ten DREAMS countries; no eligible studies from Lesotho were identified. Directly observed HIV incidence rates were lowest among females aged 13–19 years in Kumi, Uganda (0·38 cases per 100 person-years); and directly observed HIV incidence rates were highest in KwaZulu-Natal, South Africa (7·79 per 100 person-years among females aged 15–19 years, and 8·63 in those aged 20–24 years), among fishing communities in Uganda (12·40 per 100 person-years in females aged 15–19 years and 4·70 in those aged 20–24 years), and among female sex workers aged 18–24 years in South Africa (13·20 per 100 person-years) and Zimbabwe (10·80). In pooled rates from the general population studies, the greatest sex differentials were in the youngest age groups—ie, females aged 15–19 years compared with male peers in both southern African (pooled relative risk 5·94, 95% CI 3·39–10·44) and eastern African countries (3·22, 1·51–6·87), and not significantly different among those aged 25–29 years in either region. Incidence often peaked earlier (during teenage years) among high-risk groups compared with general populations. Since 2005, HIV incidence among adolescent girls and young women declined in Rakai (Uganda) and Manicaland (Zimbabwe), and also declined among female sex workers in Kenya, but not in the highest-risk communities in South Africa and Uganda.

**Interpretation:**

Few sources of direct estimates of HIV incidence exist in high-burden countries and trend analyses with disaggregated data for age and sex are rare but indicate recent declines among adolescent girls and young women. In some of the highest-risk settings, however, little evidence exists to suggest ART availability and other efforts slowed transmission by 2016. Despite wide geographical diversity in absolute levels of incidence in adolescent girls and young women, risk relative to males persisted in all settings, with the greatest sex differentials in the youngest age groups. To end new infections among the growing population of adolescents in sub-Saharan Africa, prevention programmes must address gender inequalities driving excessive risk among adolescent girls.

**Funding:**

This work was conducted as part of a planning grant funded by the Bill & Melinda Gates Foundation.

## Introduction

Young people, and young women in particular, have been identified as a group at disproportional risk of HIV infection. Global estimates from 2015 indicate that young people represent 34% of all new HIV infections, with adolescent girls and young women accounting for most of those.^1^ The UNAIDS' 2014 Gap Report highlights the particularly high burden of HIV among young women in sub-Saharan Africa, where 80% of all young women living with HIV infection reside.^2^

The high levels and unequal distribution of HIV infection among young people have prompted a focus on adolescents as a target population for HIV prevention. In December, 2014, for example, the US President's Emergency Plan for AIDS Relief (PEPFAR) set bold and urgent HIV prevention and treatment targets, including the reduction of HIV incidence among adolescent girls and young women by 40% within 2 years. The so-called DREAMS Partnership, led by PEPFAR, the Bill & Melinda Gates Foundation, and Girl Effect, seeks to achieve this reduction through scale-up of interventions targeting the root causes of vulnerability to HIV acquisition in adolescent girls and young women, including biological, behavioural, social, and structural sources. A core package of interventions aims to promote determined, resilient, empowered, AIDS-free, mentored, and safe adolescent girls and young women in ten sub-Saharan African countries (Kenya, Lesotho, Malawi, Mozambique, South Africa, Swaziland, Tanzania, Uganda, Zambia, and Zimbabwe) that together account for more than half of all new HIV infections globally in adolescent girls and young women.^3^, ^4^

**Research in context**

**Evidence before this study**

Young women have been identified as a population group at particular risk for HIV infection. Epidemiology reports of the UN Programme on HIV/AIDS, based on surveys and mathematical modelling of HIV prevalence data in high-burden countries, consistently show prevalence increasing quickly between the ages of 15 and 24 years, more steeply in women than in men. Declines in HIV incidence have been reported among adult populations in numerous settings since widespread roll-out of antiretroviral therapy (ART).

**Added value of this study**

Compiling direct estimates of HIV incidence among young people—from published studies and unpublished data—has revealed a heterogeneous body of evidence to understand trends among young people since ART availability. In the decade following ART roll-out, we found indications that absolute levels of incidence among adolescent girls and young women had declined in Rakai (Uganda), Manicaland (Zimbabwe), and nationally in South Africa, but not yet in the highest-burden settings within South Africa—where rates among the general population of young women matched those among the highest-risk populations elsewhere, such as female sex workers in Zimbabwe and bar workers in Tanzania. Rates of HIV also did not decline among adolescent girls and young women in fishing communities in Uganda. Among high-risk groups, this review shows that incidence can peak at an earlier age than in general populations—risk is commonly higher among females aged 15–19 years than those aged 20–24 years in studies sampling sex workers, bar workers, or pregnant women. One analysis of time trends in incidence among a high-risk population—sex workers in Mombasa—showed benefits from ART coverage and other initiatives. Gender disparities in risk persisted since ART availability. HIV risk was higher among females aged 15–19 years than among male peers in all studies, settings, and timepoints; in pooled analyses, this increased up to six times higher for females in the southern African region and three times higher for females in eastern Africa.

**Implications of all the available evidence**

Our review shows emerging evidence for recent declines in HIV incidence among adolescent girls and young women but the availability of ART and other interventions might be insufficient or slow to halt HIV infections among young women in the highest risk groups and settings, or rectify the relative risk for female adolescents. The persistent gender and age factors that drive transmission dynamics must be directly addressed to accelerate declines and achieve epidemic control goals. Supporting young adult men through testing, prevention, and treatment cascades must be part of the solution.

In another recent initiative—All In to End Adolescent AIDS—UNICEF and global partners aim to reduce new HIV infections among adolescents (10–19 years) by 75% by 2020, and end the AIDS epidemic among adolescents by 2030. Decisions about such investments have been based largely on HIV prevalence data and modelled incidence estimates, which are more readily available than data from observed incidence. Although prevalent cases will often represent or proxy recent infection among young people, because of recent sexual debut, prevalence estimates will also include those infected vertically.^5^ The number of such children is unknown, but this number is often substantial and rising with increased survival because of scale-up of antiretroviral therapy (ART).

Directly observed estimates of HIV incidence can be more useful than prevalence data in identifying the timing of new infections, and consequently the windows of opportunity and the populations and places of highest risk, for maximum impact of prevention efforts. Incidence estimates can also reveal changes in HIV transmission over time, such as the past decade in which mass scale-up of ART and prevention of mother-to-child transmission changed the context of risk. As more sexual partners reach viral suppression, transmission and incidence should decline as ART coverage increases, and indeed, evidence is emerging of declining incidence at global, regional, and national levels.^6^ Yet, it is not clear whether new infections are slowing among young people in the highest prevalence settings. ART adherence among male sexual partners of adolescent girls and young women is not well known, and among young people themselves, uptake of HIV testing and coverage of ART are low and AIDS-related deaths remain high, relative to all other age groups.^7^, ^8^ The most recent review of HIV incidence studies summarised estimates published through 2007 and identified scant data from direct estimation of HIV incidence in high-prevalence countries, with few estimates for young people specifically.^9^

In light of the aforementioned, we sought to identify more recent estimates of HIV incidence, for better understanding of adolescent girls' and young women's risk in the time since ART scale-up—considered the third phase of the global HIV pandemic^10^—and to help inform prevention efforts that target young people.

## Methods

### Search strategy and selection criteria

We did a systematic review and meta-analysis. We sought to summarise recent levels and trends in HIV incidence among adolescent girls and young women in the ten countries selected in 2015 for DREAMS investment: Kenya, Lesotho, Malawi, Mozambique, South Africa, Swaziland, Tanzania, Uganda, Zambia, and Zimbabwe. Articles with HIV incidence estimates were identified through searches of databases for peer-reviewed published papers, and through contact with authors of eligible studies for data further disaggregated by age and sex.

Our inclusion criteria consisted of the following: timeframe (HIV incidence estimates based on serological data collected from 2005 [a proxy for roll-out of ART and prevention of mother-to-child transmission] to 2015 [proxy baseline for DREAMS implementation, which began in 2016]), location (study done in a DREAMS country), age (15–24 years included in the study sample), population (includes data for adolescent girls and young women [their male counterparts also considered]), study design (studies with collection of serological samples, either with repeat HIV testing through prospective designs or use of assays for estimates of recent HIV infection; incidence estimates based on mathematical modelling were not included), and outcome of interest (ie, HIV incidence). We only included articles published in English.

We systematically searched MEDLINE, Embase, Web of Science, Global Health, and CINAHL using the search terms “HIV incidence”, “adolescents”, and “girls and young women”. Medical subject headings were used and truncation, synonyms, and alternative spelling were added to the search. In addition to the database searches, we searched the citations from key papers identified for inclusion in the review.

KdG, IB, and CT applied the inclusion criteria to all studies identified through the database searches. SS independently did the eligibility assessment using the inclusion criteria for 20% of the studies at the initial screening of titles, abstracts, and full texts. Assessment of agreement was undertaken at each of these stages, and any disagreement resolved by consensus after referring to the protocol. For studies identified through the search, which published directly observed HIV incidence estimates within the eligible countries and timeframe, but not by age and sex, we contacted the authors to request disaggregated estimates.

Standardised data were extracted from each study, which were study design, country, location (province or district), study period, study population, person-years at risk, number of HIV seroconversions, statistical methods for incidence estimation (where these were reported), and HIV incidence estimates reported for participants up to the age of 30 years. Incidence rate was converted to the number of new HIV infections per 100 person-years, if a different unit was presented by authors. We reviewed reported HIV incidence rates by sex, age range, setting, and time or year. We used checklists from the Critical Appraisal Skills Programme^11^ and the Joanna Briggs Institute^12^ (for cross-sectional studies) to appraise the methodology of included studies.

### Data analysis

Analysis of estimates from general population studies consisted of three components: construction of forest plots, calculation of pooled HIV incidence rates, and meta-regression analysis. First, we constructed forest plots to summarise HIV incidence rates and 95% CIs by sex and age, and stratified by region. When CIs were not reported, we derived them using exact methods based on a Poisson distribution. Second, we used a random-effects meta-analysis technique^13^ to calculate pooled estimates of sex and age-specific incidence rates, stratified by region, from studies based on prospective designs (excluding cross-sectional, assay-based studies), when the number of HIV events and risk measured in person-years by sex and age were available. Meta-analysis models were applied by using natural logarithm HIV incidence rates and a corresponding SE. Studies reporting zero HIV events posed a challenge for natural logarithm function.^14^ For these studies, we added 0·5 to the number of HIV events and person-years of follow-up as correction methods.^15^

In terms of publication bias and small study effects, we neither constructed funnel plots (as this method is not recommended for small sample sizes of less than ten studies^16^) nor applied Begg's or Egger's tests given inadequate numbers of estimates for detection.^17^, ^18^ Since HIV incidence is expected to vary from one setting to another, we did not report a statistical test for heterogeneity. As a sensitivity analysis for southern Africa region, where multiple studies were from the same surveillance site, we calculated pooled estimates both with and without estimates from that site. Lastly, we used meta-regression to assess differences in pooled incidence rates by age and sex, within regions.

We did all statistical analyses using Stata (version 15.0).

### Role of the funding source

The funders of the study had no role in study design, data collection, data analysis, data interpretation, or writing of the report. The corresponding author had full access to all the data in the study and had final responsibility for the decision to submit for publication.

## Results

**Figure 1:**
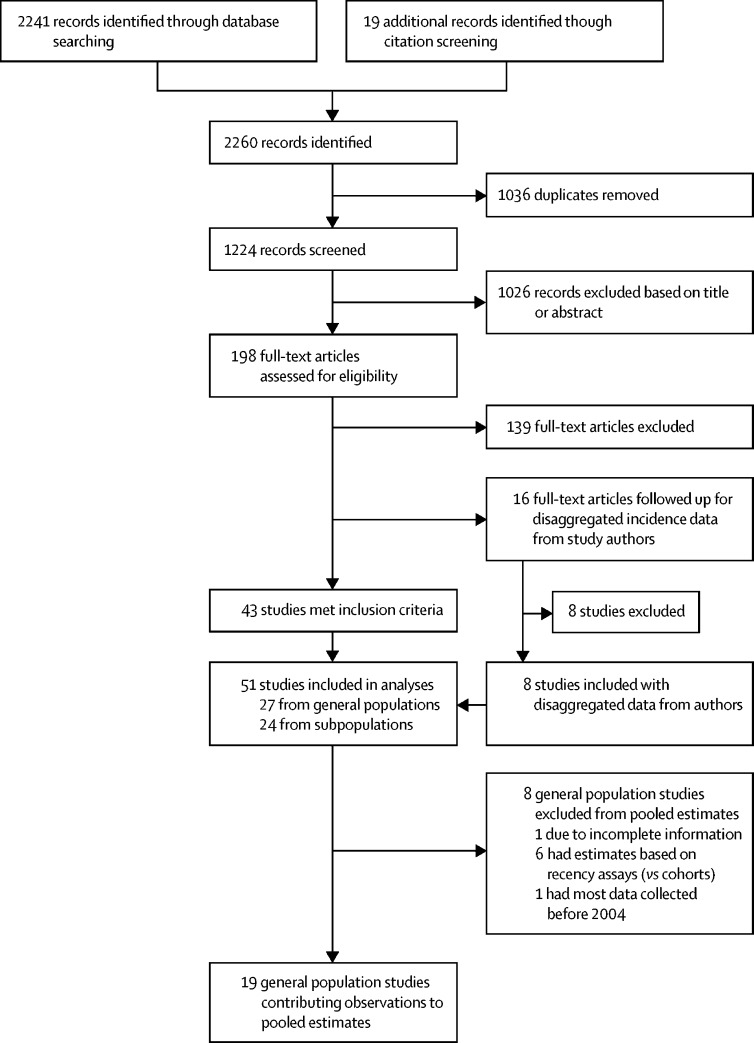
Flow of information through the search and screening phases of the review

Figure 1 summarises the flow of information through each phase of the systematic review. 2241 records were identified through the database searches and 19 through citation screening. After duplicates were removed, 1224 studies remained, of which a further 1026 were excluded based on their title or abstract. In total, we examined the full text of 198 studies. Of these studies, 139 were excluded based on inadequate incidence reporting or study design. 43 studies met all inclusion criteria identified in the original protocol and reported age-specific and sex-specific estimates for HIV incidence. 16 additional studies did not report HIV incidence separately for 15–24-year-old females (but were eligible otherwise), and age-disaggregated and sex-disaggregated data were sought through contact with study authors. This follow-up yielded data from eight more studies, for a total of 51 studies included in this systematic review. The appendix lists the location and other details of eligible studies for which disaggregated data were not available.

The 51 studies included were done in nine of the ten DREAMS countries: Kenya (n=4), Malawi (n=1), Mozambique (n=4), South Africa (n=21), Swaziland (n=1), Tanzania (n=2), Uganda (n=8), Zambia (n=1), Zimbabwe (n=4), and multicountry studies (n=5). No eligible studies from Lesotho were identified. 27 studies reported incidence estimates from general population-based samples (table 1)—ie, census-based surveillance or random samples from household sampling frames. The other 24 studies reported estimates from subpopulations of young people, on the basis of high-risk sexual behaviour or venue-based samples drawn from schools, bars, guesthouses, clinics, or fishing villages (table 2).

**Table 1:** HIV incidence estimates among young women and men in samples representing the general population

	**Country**	**Location, region, or setting**	**Study design (measure of HIV incidence)**	**Year**	HIV incidence per 100 PYs (95% CIs)*					
					Females			Males		
					Age 15–19 years	Age 20–24 years	Age 25–30 years	Age 15–19 years	Age 20–24 years	Age 25–30 years
Birdthistle et al (2018)^19^ and Borgdorff et al (2018)^20^	Kenya	Gem subcounty, Siaya county	Prospective cohort (direct estimates)	2011–16	0·43 (2·96–5·91); n=35;PYs=8236	1·12 (0·80–1·52); n=40; PYs=3574	0·96 (0·73–1·25)†; n=56; PYs=5800	0·32 (0·19–0·51); n=17;PYs=5300	Included in previous estimate	1·07 (0·71–1·57)†; n=27;PYs=2500
Blaizot et al (2017)^21^	Kenya	Ndhiwaza subcounty, Siaya county (former province of Nyanza)	Cross-sectional survey (test for recent infection; estimates from assay measurements)	2012	BioRad avidity assay: 2·07 (0·85–3·29); n=18; N=184; limiting antigen avidity enzyme immunoassay 2·50 (1·10–4·50); n=11; N=184	Included in previous estimate	BioRad avidity assay: 1·90 (0·00–3·90)†; n=16; N=359; limiting antigen avidity enzyme immunoassay: 2·40 (0·20–5·10)†; n=8; N=359	BioRad avidity assay: 0·25 (0·00–0·73); n=2; N=25; limiting antigen avidity enzyme immunoassay: 0·30 (0·00–2·70); n=1; N=25	Included in previous estimate	BioRad avidity assay: 2·50 (0·20–4·60)†; n=9; N=121; limiting antigen avidity enzyme immunoassay: 0·96 (0·00–2·79)†; n=2; N=121
Malawi Ministry of Health (2017)^22^	Malawi	National	Cross-sectional (test for recent infection)	2015–16	0·40(0·04–0·77)	Included in previous estimate	0·87 (0·11–1·63)†	0·05(0·00–0·19)	Included in previous estimate	0·40 (0·00–0·91)†
Vandormael et al (2014)^23^	South Africa	uMkhanyakude district, KwaZulu-Natal	Prospective cohort (direct estimates)	2004–12	4·91 (4·48–5·39); n=451; PYs=9179	7·80 (7·19–8·46); n=583; PYs=7478	6·50 (5·66–7·45)‡; n=204; PYs=3140	0·90 (0·71–1·12); n=75; PYs=8373	3·28 (2·85–3·78); n=192; PYs=5848	4·66 (3·82–5·68)‡; n=97; PYs=2082
Harling et al (2014)^24^	South Africa	uMkhanyakude district, KwaZulu-Natal (sexually active)	Prospective cohort (direct estimates)	2003–12	7·79 (6·59–9·22)	8·63 (7·63–9·77); n=458; PYs=5913	5·63 (4·46–7·11)‡	··	··	··
Rosenberg et al (2013)^25^	South Africa	uMkhanyakude district, KwaZulu-Natal	Prospective cohort (direct estimates)	2006–11	4·37 (3·79–5·04); n=190; PYs=4344	Included in previous estimate	··	1·38(1·07–1·79); n=58; PYs=4193	Included in previous estimate	··
Tanser et al (2011)^26^	South Africa	uMkhanyakude district, KwaZulu-Natal	Prospective cohort (direct estimates)	2004–09	5·10 (4·58–5·67); n=342; PYs=6701·51	7·47 (6·33–8·76); n=152; PYs=2033·72	5·18 (3·88–6·77)‡; n=53; PYs=1023·38	··	··	··
Tanser et al (2013)^27^	South Africa	uMkhanyakude district, KwaZulu-Natal	Prospective cohort (direct estimates)	2004–11	4·43 (3·96–4·95); n=312; PYs=7036	6·49 (5·86–7·18); n=375; PYs=5776	5·51 (4·64–6·54)‡; n=131; PYs=2376	0·74 (0·56–0·99); n=48; PYs=6458	2·53 (2·10–3·05); n=109; PYs=4310	4·43 (3·50–5·61)‡; n=69; PYs=1557
Dobra et al (2017)^3^	South Africa	uMkhanyakude district, KwaZulu-Natal	Prospective cohort (direct estimates)	2004–14	5·10 (4·61–5·60); n=388; PYs=7604	9·11 (8·40–9·82); n=570; PYs=6257	7·03 (6·07–7·99)‡; n=192; PYs=2732	0·91 (0·70–1·12); n=72;PYs=7908	3·69 (3·19–4·19); n=203; PYs=5504	5·79 (4·79–6·80)‡; n=121; PYs=2088
Akullian et al (2017)^28^	South Africa	uMkhanyakude district, KwaZulu-Natal	Prospective cohort (direct estimates)	2004–15	5·6(5·3–5·9); n=1173;PYs=21 043	Included in previous estimate	3·1(2·9–3·4)§; n=615;PYs=19 863	1·7(1·5–1·9); n=296;PYs=17 915	Included in previous estimate	3·6 (3·1–4·2)†; n=184; PYs=5061
Chimbindi et al (2018)^29^	South Africa	uMkhanyakude district, KwaZulu-Natal	Prospective cohort (direct estimates)	2006–10	4·71(4·10–5·41); n=254; PYs=5395	7·62 (6·71–8·65); n=340; PYs=4462	··	··	··	··
Chimbindi et al (2018)^29^	South Africa	uMkhanyakude district, KwaZulu-Natal	Prospective cohort (direct estimates)	2011–15	4·54 (3·89–5·30); n=197; PYs=4330	7·45 (6·51–8·51); n=289; PYs=3881	··	··	··	··
Baisley et al (2018)^30^	South Africa	uMkhanyakude district, KwaZulu-Natal	Prospective cohort (direct estimates)	2006–10	··	··	··	··	3·08 (2·49–3·82); n=120; PYs=3897	4·43 (3·34–5·87)‡; n=68; PYs=1530
Baisley et al (2018)^30^	South Africa	uMkhanyakude district, KwaZulu-Natal	Prospective cohort (direct estimates)	2011–15	··	··	··	··	2·58 (2·00–3·32); n=81; PYs=3121	4·04 (3·07–5·31)‡; n=73; PYs=1793
Bärnighausen et al (2008)^31^¶	South Africa	uMkhanyakude district, KwaZulu-Natal	Prospective cohort (direct estimates)	2003–07	3·9(2·9–5·3)	5·6(4·0–8·0)	8·0(4·9–13·0)‡	1·0(0·5–1·8)	2·8(1·6–4·8)	8·7(4·8–15·8)‡
Unpublished data from Iwuji et al (2018)^32^	South Africa	uMkhanyakude district (Hlabisa subdistrict), KwaZulu-Natal	Intervention (direct estimates; control arm)	2012–15	5·54 (4·64–6·45)‖; n=144; PYs=2597	6·93(5·75–8·11); n=133; PYs=1919	6·34 (4·77–7·90)‡; n=63; PYs=994	0·61 (0·28–0·94)‖; n=13; PYs=2144	2·02 (1·19–2·84); n=23; PYs=1141	1·06 (0·21–1·90)‡; n=6; PYs=567
Shisana et al (2005)^33^	South Africa	National	Cross-sectional (test for recent infection; estimates from assay measurements)	2005	6·5 (2·3–10·7); n=61; N=2335	Included in previous estimate	7·1 (2·6–11·6)†; n=48; N=2013 (for men and women combined)	0·8 (0·0–3·4); n=9; N=1785	Included in previous estimate	7·1 (2·6–11·6)†; n=48; N=2013 (for men and women combined)
Shisana et al (2014)^7^	South Africa	National	Cross-sectional (test for recent infection; estimates from assay measurements)	2012	2·54 (2·04–3·04); N=3092	Included in previous estimate	1·62 (1·30–1·94)**; N=8857	0·55 (0·45–0·65); N=2798	Included in previous estimate	1·29 (0·91–1·67)**; N=5959
Justman et al (2017)^6^††	Swaziland	National	Prospective cohort (direct estimates)	2010–11	3·8 (2·6–5·6)‡‡; n=16·0; PYs=421·1	4·3 (3·3–5·6); n=36·4; PYs=846·5	2·0 (1·2–3·2)‡; n=10·2; PYs=510	0·8 (0·4–1·9)‡‡;n=4·0;PYs=500	1·6 (1·1–2·5); n=16·0; PYs=1000	2·6 (1·7–4·0)‡; n=16·9; PYs=650
Unpublished data from Geis et al (2011)^34^§§	Tanzania	Mbeya	Prospective cohort (direct estimates)	2005–06	0¶¶	2·60(1·51–4·47)	0·26 (0·04–1·84)‡	0¶¶	1·36 (0·57–3·27)	0·72 (0·18–2·88)‡
Okiria et al (2014)^35^††‖‖	Uganda	Kumi district (rural)	Prospective cohort (direct estimates)	2006–08	0·38 (0·19–0·66)***; n=11; PYs=2894·7	··	0·68 (0·44–1·01)‡; n=25; PYs=3676·5	0·26 (0·07–0·66)***; n=4; PYs=1538·5	··	0·28 (0·09–0·65)‡; n=5; PYs=1785·7
Biraro et al (2013)^36^††	Uganda	Masaka	Prospective cohort (direct estimates)	1990–2007	0·57 (0·44–0·74)***; n=58; PYs=10 104	1·19 (0·91–1·53); n=59; PYs=4962	0·95 (0·68–1·30)‡; n=39; PYs=4108	0·09 (0·04–0·16)***; n=10; PYs=11 261	0·79 (0·55–1·09); n=36; PYs=4554	1·35 (0·99–1·80)‡; n=46; PYs=3415
Unpublished data from Grabowski et al (2017)^37^	Uganda	Rakai	Prospective cohort (direct estimates)	2005–11	1·03 (0·64–1·57); n=19; PYs=1840	1·47 (1·12–1·90); n=55; PYs=3730	1·47 (1·15–1·84)‡; n=70; PYs=4772	0·30 (0·12–0·60); n=6; PYs=2023	0·90 (0·61–1·28); n=28; PYs=3107	1·73 (1·31–2·23)‡; n=55; PYs=3173
Unpublished data from Grabowski et al (2017)^37^	Uganda	Rakai	Prospective cohort (direct estimates)	2011–16	0·59(0·31–1·01); n=11; PYs=1857	1·53(1·13–2·01); n=47; PYs=3077	1·12 (0·81–1·50)‡; n=41; PYs=3664	0·16 (0·05–0·38); n=4; PYs=2466	0·40 (0·21–0·68); n=12; PYs=2973	1·26 (0·89–1·72)‡; n=55; PYs=2783
Santelli et al (2013)^38^	Uganda	Rakai (sexually experienced)	Prospective cohort (direct estimates)	1998–2008	1·49 (1·06–2·04); n=39; PYs=2614	1·38 (1·13–1·66); n=109; PYs=7907	··	0·36 (0·14–0·73); n=7; PYs=1969	1·02 (0·76–1·35); n=49; PYs=4803	··
Santelli et al (2015)^39^††	Uganda	Rakai (sexually experienced)	Prospective cohort (direct estimates)	2006–09	0·63 (0·20–1·47); n=5; PYs=793·7	1·31 (0·82–1·98); n=22; PYs=1679·4	··	0·22 (0·03–0·79); n=2; PYs=909·1	0·79 (0·38–1·45); n=10; PYs=1265·8	··
Santelli et al (2015)^39^††	Uganda	Rakai (sexually experienced)	Prospective cohort (direct estimates)	2008–11	0·23 (0·03–0·83); n=2;PYs=869·6	1·55(0·95–2·39); n=20; PYs=1290·3	··	0·19 (0·02–0·69); n=2; PYs=1052·6	1·23 (0·69–2·03); n=15; PYs=1219·5	··
Unpublished data from Ruzagira et al (2011)^14^	Uganda	Masaka	Prospective cohort (direct estimates)	2004–07	0;n=0; PYs=64·1	0;n=0; PYs=206·8	2·0(0·8–4·8)‡; n=5; PYs=252·1	0;n=0;PYs=26	2·5(0·8–7·7); n=3; PYs=121·4	2·2(0·7–6·8)‡; n=3;PYs=139·5
Zambia Ministry of Health (2017)^40^	Zambia	National	Cross-sectional (test for recent infection; estimates from assay measurements)	2015–16	1·07(0·52–1·62)	Included in previous estimate	1·16 (0·46–1·86)†	0·08 (0·00–0·25)	Included in previous estimate	0·25 (0·00–0·63)†
Unpublished data from Schaefer et al (2017)^41^	Zimbabwe	Manicaland	Prospective cohort (direct estimates)	2003–13	0·99 (0·71–1·38); n=35; PYs=3530·20	1·62 (1·26–2·08); n=62; PYs=3830·1	1·45 (1·12–1·88)‡; n=57; PYs=3930·9	0·26 (0·15–0·46); n=12; PYs=4557·3	0·83 (0·55–1·26); n=22; PYs=2642·3	1·47 (1·04–2·06)‡; n=22; PYs=2250·2
Unpublished data from Schaefer et al (2017)^41^	Zimbabwe	Manicaland	Prospective cohort (direct estimates)	2004–08	1·94(1·50–2·51); n=59; PYs=3039·8	Included in previous estimate	··	0·93 (0·64–1·36); n=27; PYs=3895·0	Included in previous estimate	··
Unpublished data from Schaefer et al (2017)^41^	Zimbabwe	Manicaland	Prospective cohort (direct estimates)	2006–11	0·72 (0·46–1·14); n=19; PYs=2625·4	Included in previous estimate	··	0·15 (0·06–0·39); n=4; PYs=2740·5	Included in previous estimate	··
Unpublished data from Schaefer et al (2017)^41^	Zimbabwe	Manicaland	Prospective cohort (direct estimates)	2009–13	1·12(0·72–1·76); n=19; PYs=1695·0	Included in previous estimate	··	0·19 (0·06–0·60); n=3; PYs=1564·2	Included in previous estimate	··
Zimbabwe MOHCC (2017)^42^	Zimbabwe	National	Cross-sectional (test for recent infection; estimates from assay measurements)	2015–16	0·53(0·13–0·93)	Included in previous estimate	1·11 (0·41–1·80)†	0·14 (0·00–0·37)	Included in previous estimate	0·48 (0·00–1·05)†

PYs=person-years. Included in previous estimate=data for age groups 15–19 years and 20–24 years are combined into one estimate (as given in adjacent cell). MOHCC=Ministry of Health and Child Care.

* Cases (n) and PYs are shown when available. Total sample size (N) is given when PYs were not available.

† Up to age 34 years.

‡ Up to age 29 years.

§ Up to age 49 years.

¶ n=170; PYs=5253 for all men and women aged 15–34 years.

‖ Between ages 16 years and 19 years.

** Older than 25 years.

†† PYs or 95% CIs, or both, were calculated from data in study; CIs were calculated using exact Poisson CIs.

‡‡ Between ages 18 years and 19 years.

§§ n=101; PYs=7471 for study incorporating data from 2003 to 2004 (not included here) and 2005 to 2006 (included here).

¶¶ Between ages 17 years and 19 years.

‖‖ Data from a home-based HIV counselling and testing programme offered community wide.

*** Between ages 13 years and 19 years.

**Table 2:** HIV incidence estimates among subpopulations of young women and men

	**Country**	**Location, region, or setting**	**Subpopulation**	**Study design (measure of HIV incidence)**	**Year**	HIV incidence per 100 PYs (95% CIs)*					
						Females			Males		
						Age15–19 years	Age 20–24 years	Age 25–30 years	Age15–19 years	Age 20–24 years	Age 25–30 years
Mdodo et al (2016)^43^†	Kenya	Kisumu	High-risk women (recruited from social and clinic venues and respondent-driven sampling)	Prospective cohort (direct estimates)	2010–11	··	4·21(1·36–9·82);n=5; PYs=119	··	··	··	··
Unpublished data from Masese et al (2015)^44^ and McClelland et al (2015)^45^	Kenya	Mombasa district	Female sex workers	Prospective cohort (direct estimates)	2005–12	0 (0–1·99);n=0;PYs=185·4	0·4 (0·1–0·9);n=5;PYs=1291·5	0·6 (0·3–1·0)‡; n=11;PYs=1919·7	··	··	··
De Schacht et al (2014)^46^	Mozambique	Southern Mozambique (Gaza and Maputo provinces)	Pregnant women	Prospective cohort (direct estimates)	2008–11	4·92(2·65–9·15)§	3·39(2·08–5·53)	3·49(1·88–6·49)‡	··	··	··
Dube et al (2014)^47^	Mozambique	Beira (Sofala Province)	High-risk women (reporting >2 partners in the last month)	Prospective cohort (direct estimates)	2009–12	··	7·1 (4·3–11·1)¶; n=19;PYs=266·5	6·0(1·2–17·7)‡; n=3; PYs=49·6	··	··	··
Feldblum et al (2014)^48^‖	Mozambique	Chokwe (Gaza Province)	High-risk women (recruited from bars, guesthouses, similar facilities, and secondary schools)	Prospective cohort (direct estimates)	2010–12	··	4·8 (2·5–8·3)¶	4·5(1·2–11·4)‡	··	··	··
Viegas et al (2015)^49^	Mozambique	Maputo	Youth aged 18–24 years at a youth clinic	Prospective cohort (direct estimates)	2009–11	··	1·49 (0·81–2·50)¶; n=14; PYs=940·23	··	··	0 (0·00–1·27)¶; n=0; PYs=289·56	··
Abdool Karim et al (2011)^50^	South Africa	KwaZulu-Natal (Vulindlela and central Durban)	Women aged 14–30 years from family planning and STI clinics	Prospective cohort (direct estimates)	2004–07	4·2 (2·1–7·6)**; n=11;PYs=260·7	8·0 (4·9–12·3); n=20;PYs=249·5	8·7(3·8–17·2);n=8;PYs=91·8	··	··	··
Ramjee et al (2012)^51^††	South Africa	KwaZulu-Natal (rural Umkomaas, Botha's Hill, Durban, semi-rural Tongaat, Verulam and rural district of Hlabisa)	Non-pregnant women (recruited through clinics, home visits, community meetings, word of mouth)	Intervention (data from three trials)	2002–05	10; N=965	Included in previous estimate	6·0‡‡; N=774	··	··	··
Jewkes et al (2008)^52^†	South Africa	Eastern Cape	Youth aged 15–26 years attending school (control arm only)	Intervention(direct estimates)	2003–06	6·95 (5·40–8·81)§§; n=68;PYs=978·4	Included in previous estimate	··	1·29 (0·69–2·21)§§; n=13;PYs=1007·8	Included in previous estimate	··
Naicker et al (2015)^53^	South Africa	KwaZulu-Natal, Durban	≥18 years identifying as female sex workers or reporting ≥3 partners in 3 months before recruitment	Prospective cohort (direct estimates)	2004–07	··	13·20 (6·59–23·62)¶; n=11;PYs=83·32	4·58 (2·50–7·68)¶¶; n=14;PYs=305·90	··	··	··
Fatti et al (2017)^54^	South Africa	KwaZulu-Natal, Durban	Pregnant and post-partum women recruited from health facility (intervention group; no control)	Intervention(direct estimates)	2013–15	0·00 (0·00–1·48)**; n=0;PYs=248·9	1·63(0·68–3·93);n=5;PYs=305·4	2·19 (0·98–4·88)¶¶; n=6;PYs=273·9	··	··	··
Pettifor et al (2016)^55^†	South Africa	Mpumalanga province (Bushbuckridge subdistrict)	Girls aged 13–20 years enrolled in school grades 8–11 (control arm)	Intervention(direct estimates)	2011–15	2·0 (1·5–2·7)‖‖; n=48;PYs=2400	··	··	··	··	··
Skoler-Karpoff et al (2008)^56^	South Africa	KwaZulu-Natal (Isipingo), Gauteng (Soshanguve), and Western Cape (Gugulethu)	Sexually active, HIV-negative women aged ≥16 years (recruited from clinics and community venues; placebo group)	Intervention(direct estimates)	2004–07	0·20 (0·09–0·32)***; n=13; N=120	0·09 (0·07–0·12)†††;n=60;N=965	0·06(0·04–0·07);n=78;N=1909	··	··	··
Byrne et al (2016)^57^†	South Africa	KwaZulu-Natal, Umlazi	HIV-negative, 18–23-year-old women (referred by community organisations and outreach events)	Prospective cohort (direct estimates)	2012–15	··	7·43 (4·59–11·36)‡‡‡; n=24; PYs=323	··	··	··	··
Watson-Jones et al (2009)^58^†	Tanzania	Northwestern	High-risk women (recruited from bars, guesthouses, and similar facilities)	Intervention(direct estimates)	2003–05	10·26 (4·43–20·21)§§§; n=8;PYs=78	4·88 (2·79–7·92); n=16;PYs=328	5·13(3·29–7·63)‡; n=24;PYs=468	··	··	··
Unpublished data from Abaasa et al (2016)^59^	Uganda	Masaka district	Adults at high risk for HIV in fishing communities¶¶¶	Prospective cohort (direct estimates)	2012–15	12·4(4·0–38·3);n=3; PYs=24·3	4·7(1·2–18·8); n=2; PYs=42·5	13·8(5·7–33·2)‡; n=5; PYs=36·2	19·2(6·2–59·6); n=3; PYs=15·6	2·7(0·7–10·8); n=2; PYs=74·0	3·9 (1·3–12·1)‡; n=3; PYs=76·7
Unpublished data from Seeley et al (2012)^60^	Uganda	Masaka, Wakiso, and Mukono districts	Adults from fishing communities	Prospective cohort (direct estimates)	2009–11	13·8(6·9–27·5);n=8;PYs=58·2	4·6(2·2–9·6);n=7;PYs=152·3	2·3(0·7–7·0)‡; n=3;PYs=132·0	2·3(0·3–16·2); n=1;PYs=43·7	8·0(4·5–14·0); n=12; PYs=150·5	6·6 (3·7–11·6)‡; n=12; PYs=182·0
Hargreaves et al (2016)^61^	Zimbabwe	Multiple sites across national network of sex workers	Female sex workers	Cohort analysis (direct estimates)	2009–14	10·8 (8·1–16·1)‖‖‖; n=27;PYs=250	Included in previous estimate	10·7 (8·1–16·1)****; n=32;PYs=300	··	··	··
Munjoma et al (2010)^62^†	Zimbabwe	Harare	Pregnant women	Prospective cohort (direct estimates)	2002–08	2·9(0·6–8·7); n=15; PYs=517·2	1·7(0·3–4·7); n=16; PYs=941·2	1·0(<0·1–5·4)‡; n=5; PYs=500	··	··	··
Van Damme et al (2012)^63^†	Kenya; South Africa; Tanzania	Bondo; Bloemfontein and Pretoria; Arusha	18–35-year-old women considered to be at increased risk of HIV (control arm)	Intervention(direct estimates)	2009–11	··	5·9(3·7–8·9)¶; n=23; PYs=389·8	3·8(2·0–6·6)‡‡; n=12; PYs=316·8	··	··	··
Baeten et al (2016)^64^	Malawi, South Africa, Uganda, Zimbabwe	··	HIV-negative, non-pregnant, sexually active women aged ≥18 years (control group)	Intervention(direct estimates)	2012–15	5·4 (3·2–8·4)††††; n=44; N=451	6·1 (4·3–8·3)‡‡‡‡; n=51; N=752	3·0(2·0–4·4)§§§§; n=44; N=1192	··	··	··
Balkus et al (2016)^65^†	Malawi; South Africa; Zambia; Zimbabwe	Blantyre and Lilongwe; Durban and Hlabisa; Lusaka; Harare and Chitungwiza	HIV-negative, non-pregnant, sexually active (vaginal intercourse at least once in past 3 months) women aged ≥18 years (all arms combined)	Intervention(direct estimates)	2005–08	··	4·76 (3·62–6·16)¶; n=58; PYs=1218	3·46 (2·55–4·59)¶¶¶¶; n=48; PYs=1388	··	··	··
Nel et al (2016)^66^†	South Africa and Uganda	··	Healthy, sexually active women aged 18–45 years (control arm)	Intervention(direct estimates)	Up to 2015	8·2 (4·8–13·1)††††; n=17; PYs=207·3	··	5·5(3·9–7·5)‖‖‖‖; n=39; PYs=709·1	··	··	··
Padian et al (2007)^67^†	South Africa; Zimbabwe	Durban and Johannesburg; Harare	Sexually active (average of 4 sexual acts per month) women aged 18–49 years; recruited from family planning, well baby and general health clinics, and from community-based organisations through printed media and radio (control arm)	Intervention(direct estimates)	2003–06	··	5·1(4·0–6·4)¶; n=71; PYs=1394	3·6(2·7–4·6); n=57; PYs=1592	··	··	··

STI=sexually transmitted infection. PYs=person-years. Included in previous estimate=data for age groups 15–19 years and 20–24 years are combined into one estimate (as given in adjacent cell).

* Cases (n) and PYs are shown when available. Total sample size (N) is given when PYs were not available.

† PYs or 95% CIs, or both, were calculated from data in study; CIs were calculated using exact Poisson CIs.

‡ Up to age 29 years.

§ Between ages 18 years and 19 years.

¶ Between ages 18 years and 24 years.

‖ n=17 and PYs=373·1 for whole sample.

** From age 14 years.

†† n=211 and N=2523 for whole cohort.

‡‡ Up to age 34 years.

§§ Between ages 15 years and 26 years.

¶¶ Older than 25 years.

‖‖ From age 13 years.

*** Between ages 16 years and 18 years.

††† Between ages 19 years and 24 years.

‡‡‡ Between ages 18 years and 23 years.

§§§ Between ages 16 years and 19 years.

¶¶¶ High-risk individuals comprised self-reported sexually transmitted infections, unprotected sex with more than one partner, use of recreational drugs, weekly alcohol use, and frequent travel.

‖‖‖ Between ages 12 years and 25 years.

**** Between ages 26 years and 35 years.

†††† Between ages 18 years and 21 years.

‡‡‡‡ Between ages 22 years and 26 years.

§§§§ Between ages 27 years and 45 years.

¶¶¶¶ Up to 56 years.

‖‖‖‖ Between ages 22 years and 45 years.

Most estimates were drawn from prospective studies (45 [88%] of 51 studies), with cohort data from observational research or intervention trials, while six (12%) studies estimated recent HIV infection among HIV-positive samples from cross-sectional surveys.^7^, ^21^, ^22^, ^33^, ^40^, ^42^ The cohort studies estimated incidence from repeated observations, most often imputing the timing of seroconversion from the midpoint or a random-point between the latest-negative and earliest-positive antibody test dates. Details of statistical methods for estimation were not always available or consistently reported across studies.

Nationally representative estimates were available for five countries. In Swaziland, estimates were drawn from a national cohort, whereas in the four other countries, recent HIV infection among HIV-positive samples was estimated from cross-sectional surveys. In 2015–16, Population Health Impact Assessments (PHIA) in Malawi, Zambia, and Zimbabwe combined HIV-1 limiting antigen enzyme immunoassay with a viral load of more than 1000 copies per mL and an optical density of 1·5 or less as a classification for a recent HIV infection (as per WHO's recommended algorithm).^68^ Two nationally representative surveys in South Africa estimated recent infection with differing methodologies: the earliest survey in 2005 used BED capture enzyme immunoassay, and confirmatory specimens with a cutoff optical density of 0·8 were considered to be a recent HIV infection;^33^ and the 2012 study applied the WHO recommended algorithm.^7^, ^69^

Other population-based studies were in HIV surveillance systems with large (often district-wide), open community cohorts, in which members can leave or be added over time, in Siaya, Kenya (n=1),^19^, ^20^ Masaka, Uganda (n=2),^14^, ^36^ Rakai, Uganda (n=3),^37^, ^38^, ^39^ uMkhanyakude, South Africa (n=11),^3^, ^23^, ^24^, ^25^, ^26^, ^27^, ^28^, ^29^, ^30^, ^31^, ^32^ and Manicaland, Zimbabwe (n=1).^41^ One closed (with fixed membership) general population cohort in Mbeya, Tanzania,^34^ and a district-wide home-based counselling and testing programme in eastern Uganda were also identified.^35^

The studies that sampled from groups considered to be at high-risk, or of higher risk than the general populations, included female sex workers in Zimbabwe,^61^ South Africa,^53^ and Kenya;^44^, ^45^ young women reporting multiple sexual partners or pregnant women in Mozambique^46^, ^47^, ^48^ and Zimbabwe;^62^ women recruited from bars and guesthouses in Kenya^43^ and Tanzania;^58^ and young men and women residing in fishing communities in Uganda.^59^, ^60^ A number of intervention trials (in South Africa and multicountry studies) enrolled women either from family planning and clinics for sexually transmitted infections (STIs)^50^, ^54^ or outreach events,^57^ or based on recent sexual behaviour—eg, three or more sexual partners in the past 3 months.^53^, ^56^, ^63^, ^64^, ^65^, ^66^, ^67^ Finally, three studies were sampled from settings not associated with HIV risk, such as youths attending school in South Africa's Eastern Cape^52^ or Mpumalanga province^55^ and a youth clinic in Maputo, Mozambique,^49^ and non-pregnant women in KwaZulu-Natal, South Africa.^51^

**Figure 2:**
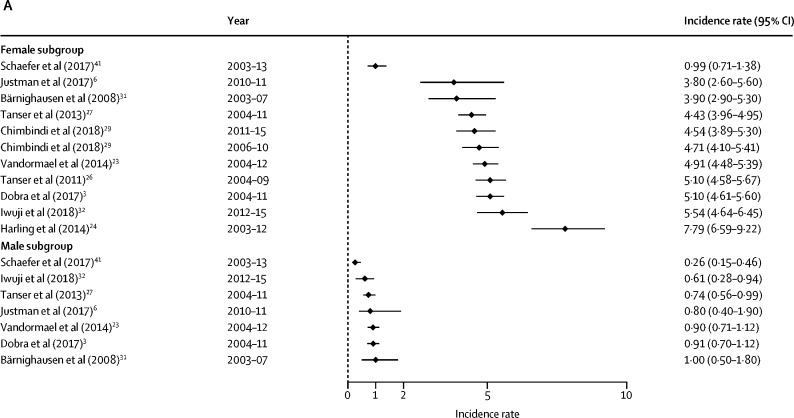

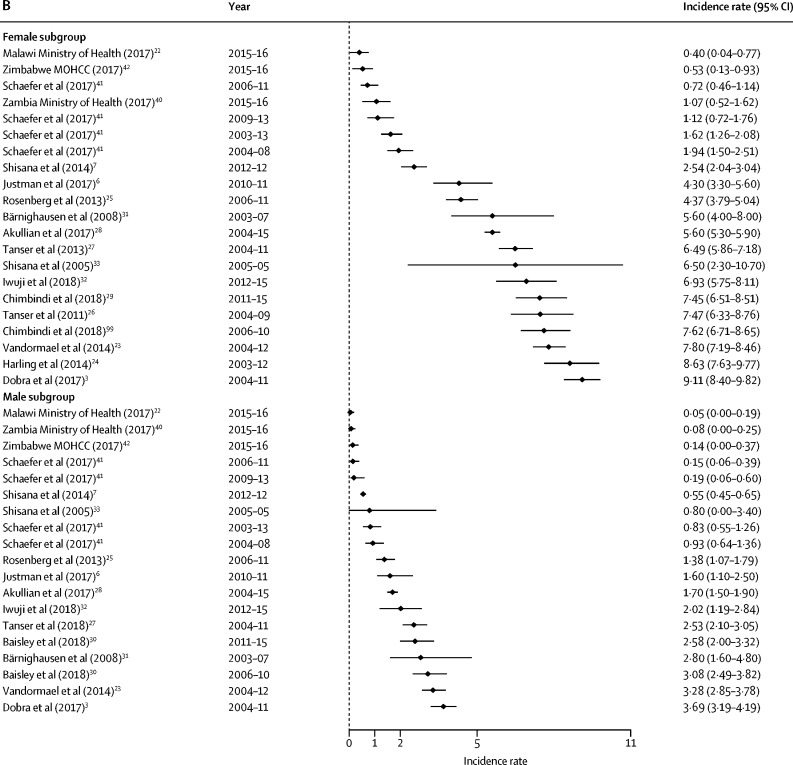

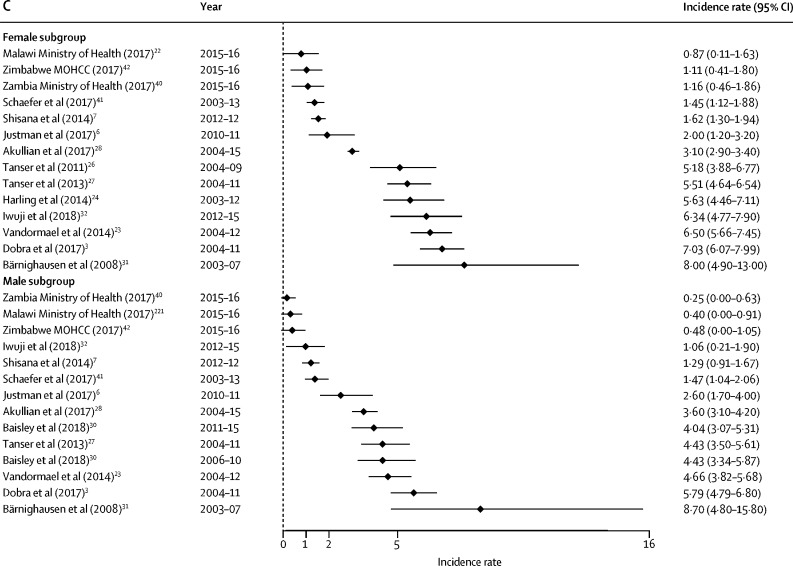
Forest plots of HIV incidence estimates for young women and men in SADC member countries Data are incidence rates (cases per 100 person-years) and error bars are 95% CIs. (A) Estimates for those aged 15–19 years. (B) Estimates for those aged 20–24 years. (C) Estimates for those aged 25–29 years. SADC=Southern African Development Community. MOHCC=Ministry of Health and Child Care.

**Figure 3:**
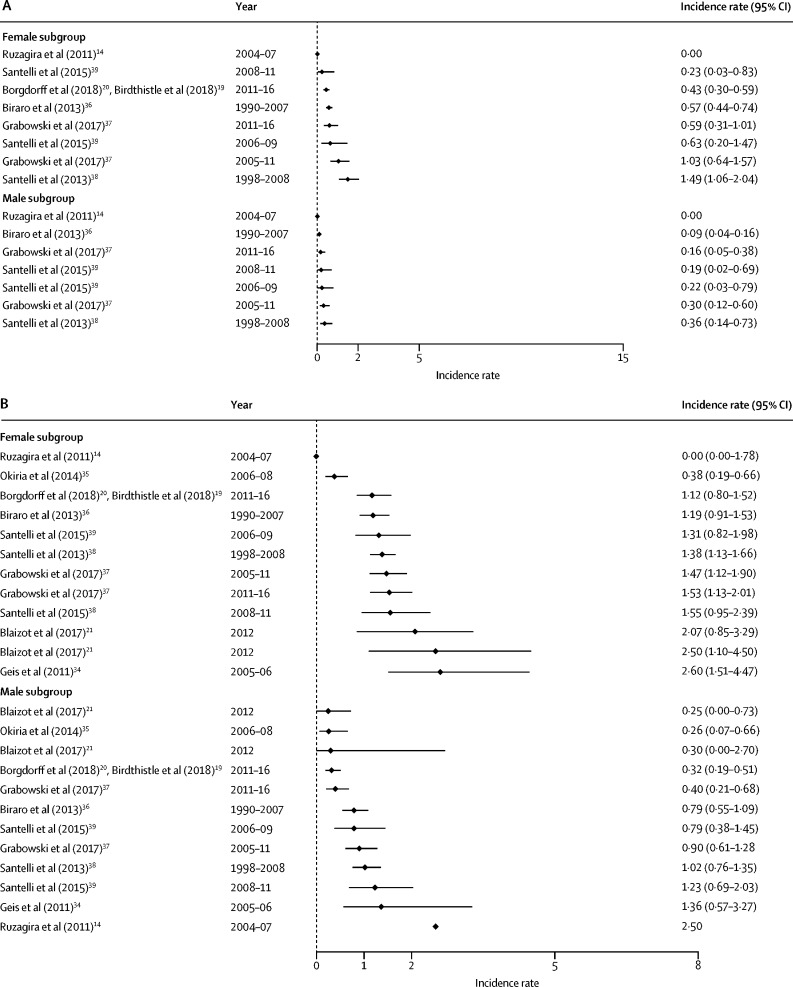

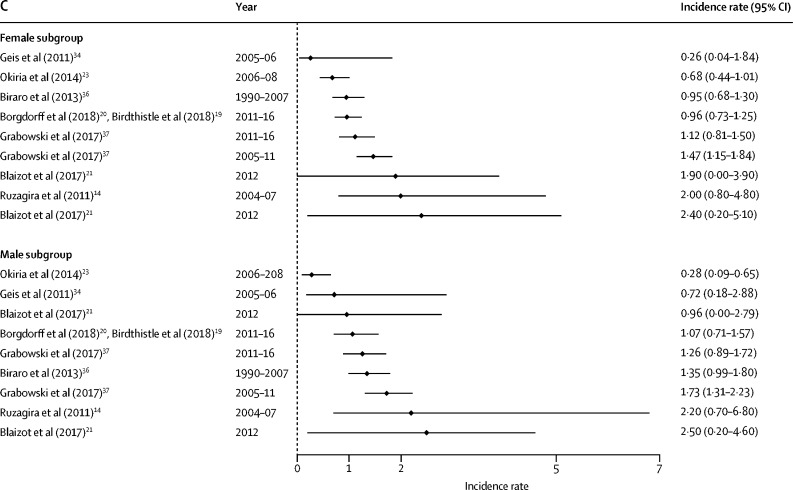
Forest plots of HIV incidence estimates for young women and men in EAC member countries Data are incidence rates (cases per 100 person-years) and error bars are 95% CIs. (A) Estimates for those aged 15–19 years. (B) Estimates for those aged 20–24 years. (C) Estimates for those aged 25–29 years. EAC=East African Community.

HIV incidence estimates from the general population studies are summarised along with study characteristics in table 1 and by age, sex, and region in Figure 2, Figure 3. National estimates of incidence were highest in South Africa (6·5 cases per 100 person-years among females aged 15–24 years in 2005,^33^ and 2·54 cases in 2012^7^) and Swaziland, where the national rates observed in 2010–11 were 3·8 cases per 100 person-years among women aged 18–19 years and 4·3 cases among those aged 20–24 years.^6^ More recently, national surveys done in 2015–16 estimated HIV incidence in Malawi to be at 0·4%, in Zambia at 1·07%, and in Zimbabwe at 0·53% among females aged 15–24 years.^22^, ^40^, ^42^

Among general populations of adolescent girls and young women in subnational areas, rates varied substantially across settings (table 1). Estimates were highest among sexually active young women in one of the earlier studies from KwaZulu-Natal (7·79 infections per 100 person-years for those aged 15–19 years; 8·63 among females aged 20–24 years between 2003 and 2012),^24^ and lowest in Uganda (eg, 0·38 cases per 100 person-years among females aged 13–19 years in the Kumi district, 2006–08).^35^ Two studies with small sample sizes observed zero seroconversions—eg, among those younger than 20 years in Mbeya (Tanzania) and Masaka (Uganda).^14^, ^34^

Among studies with high-risk populations of women, HIV incidence rates reached 12·4 by 2015 among females aged 15–19 years in Ugandan fishing communities (table 2).^59^, ^60^ Young female sex workers (younger than 25 years) experienced rates of 13·20 (95% CI 6·59–23·62) cases per 100 person-years in Durban, South Africa,^53^ and 10·8 (8·1–16·1) in Zimbabwe.^61^ High rates of 10·26 were observed among girls aged 16–19 years recruited from bars and guesthouses in Tanzania in 2003–05.^58^ Rates among females aged 14–19 years attending urban family planning and STI clinics in KwaZulu-Natal (2004–07) were lower, at 4·2 cases per 100 person-years,^50^ and a cohort of female sex workers aged 15–24 years in Kenya observed an incidence of less than 1%.^44^, ^45^ Sample sizes were small and 95% CIs were wide for many of these studies.

Three of four studies done in Mozambique reported high HIV incidence rates among subpopulations of young women, including pregnant women (4·9 among those aged 18–19 years in 2008–11),^46^ women with multiple sexual partners aged 18–24 years in Beira (7·1 per 100 person-years in 2009–12),^47^ and women aged 18–24 years in bars and guesthouses in Chokwe (4·8 in 2010–12).^48^ By contrast, incidence was lower, at 1·49, among women aged 18–24 years recruited from a youth clinic in Maputa, 2009–11.^49^

Among young males, incidence was less than 1% among those aged 15–19 years in all general population studies. The highest rates were reported among males in fishing communities—eg, previously unpublished data from fishing villages in Masaka from 2012 to 2015 showed an exceptionally high HIV incidence in adolescent boys (19·2 cases per 100 person-years; 95% CI 6·2–59·6).^59^

In studies with general populations of both males and females, HIV incidence rates among adolescent females exceeded their male peers in all settings and countries included. In South Africa and Swaziland, the rate among females aged 15–19 years was four-to-five times greater than among males. This difference narrows in the age range of those older than 25 years, when incidence among men escalates. For example, in uMkhanyakude, the large difference in incidence for males and females aged 15–19 years (5·10 per 100 person-years among females and 0·91 per 100 person-years among males)^3^ reduces as male incidence accelerates from age 20 years (7·03 per 100 person-years among females and 5·79 per 100 person-years among males).

In the general population studies, female HIV incidence rates rose between the ages 15–19 years, and typically peaked among those aged 20–24 years before declining or stabilising from age 25 years. In uMkhanyakude, for example, multiple studies show that incidence rates were highest among females aged 20–24 years and decline thereafter.^3^, ^23^, ^24^, ^26^, ^27^ In the same communities, rates accelerated later among young men; typically remaining low (<1%) among teenagers before increasing through their twenties.

By contrast, among high-risk populations, HIV incidence rates were commonly highest among the youngest females, peaking during adolescence (table 2). For example, in the Mozambique studies with high-risk populations, the highest rates were reported among the youngest age groups—eg, 4·92 among pregnant women aged 18–19 years versus 3·39 among those aged 20–24 years and 3·49 among those aged 25–29 years.^46^ Similarly, in northwestern Tanzania, females aged 16–19 years from bars and guesthouses experienced more than double the risk of those aged 20–29 years.^58^ Incidence rates were also exceptionally high among the youngest females (aged 15-19 years) in fishing communities in Uganda.^59^, ^60^ However, in women sampled from family planning and STI clinics in KwaZulu-Natal, rates were higher for women aged 20–29 years than for those aged 14–19 years.^50^

Few studies reported trends in HIV incidence over time, and the findings vary. Between 1999 and 2011, trends in HIV incidence by sex and age were reported in Rakai, Uganda. Among adolescent girls aged 15–19 years, incidence fluctuated between 1999 and 2004 (between 0·37% and 1·94%), and subsequently declined with each round through 2011 (p=0·006).^39^ More recently, between 2005–11 and 2011–16, rates declined from 1·03 to 0·59 among females aged 15–19 years and from 0·30 to 0·16 among males of the same age.^37^ Meanwhile, rates from Ugandan fishing communities in 2012–15 match or exceed estimates from Ugandan fishing communities in 2009–11,^59^, ^60^ suggesting that risk in fishing communities has not declined with time.

Estimates from the ongoing surveillance site in Manicaland, Zimbabwe, suggest declines among both young females and males between 2003 and 2013, with a substantial decrease between 2004–08 and 2006–11, before a slight increase again in more recent years of 2009–13.^41^

In the two national incidence surveys in South Africa (with representative cross-sectional samples), in 2005 and 2012, incidence reported among females aged 15–24 years declined from 6·5 (95% CI 2·3–10·7) to 2·54 (2·04–3·04); and among males of the same age from 0·8 (0·0–3·4) to 0·55 (0·45–0·65). However, the different methods used for estimating HIV incidence in the two surveys might preclude their comparability.^7^, ^33^ Meanwhile, there is no evidence of a decline in South Africa's highest-incidence settings by 2015. Estimates from the population-based HIV surveillance system with an open-community cohort in the uMkhanyakude district, KwaZulu-Natal, show no decline in rates between 2006–10 and 2011–15 among adolescent girls or young women^29^ or among young men aged 20–29 years.^30^ Newly obtained age-disaggregations of HIV incidence among female sex workers in Mombasa, Kenya, show that risk among those aged 15–19 years (2·9%) matched that of older women aged 25–29 years (3·0%) between 1998 and 2004. More recently, from 2005 to 2012, incidence observed in the same cohort study decreased (eg, zero among females aged 15–19 years and <1% among all older age groups), indicating reduction of risk over time among sex workers of all ages.^44^, ^45^

Of the 27 general population cohort studies, information about HIV events (directly estimated) needed for pooled sex and age calculations was available in 19 (70%) studies (table 3). The pooled rates are highest among young women in studies from the Southern African Development Community (SADC) region, and exceed 4% per year in all age groups from 15–29 years (this incidence decreases to <2% when studies from uMkhanyakude, South Africa, are excluded). Among young men, the pooled rates are also higher in SADC than the East African Community (EAC) region, and highest among those aged 25–29 years in both SADC (3·34 per 100 person-years, 95% CI 2·58–4·34) and EAC (1·32 per 100 person-years, 0·93–1·86).

**Table 3:** Pooled estimates of HIV incidence from 19 general population studies by age, sex, and region

	**Number of estimates**	**Age (years)**	**Sex**	**Region**	**Study inclusion (excluding assay-based estimates)**	**HIV incidence events per 100 person-years (95% CI)**
Dobra et al (2017),^3^ Justman et al (2017),^6^ Vandormael et al (2014),^23^ Tanser et al (2011),^26^ Tanser et al (2013),^27^ Chimbindi et al (2018)^29^*, unpublished data from Iwuji et al (2018),^32^ and unpublished data from Schaefer et al (2017)^41^	9	15–19	Female	SADC	All	4·22 (3·61–4·94)
Justman et al (2017)^6^ and unpublished data from Schaefer et al (2017)^41^	2	15–19	Female	SADC	Sensitivity analysis (excluding studies from uMkhanyakude)	1·92 (0·51–7·15)
Dobra et al (2017),^3^ Vandormael et al (2014),^23^ Tanser et al (2011),^26^ Tanser et al (2013),^27^ Chimbindi et al (2018),^29^* and Iwuji et al (2018)^32^	7	15–19	Female	SADC	Sensitivity analysis (uMkhanyakude studies only)	4·88 (4·64–5·13)
Dobra et al (2017),^3^ Justman et al (2017),^6^ Vandormael et al (2014),^23^ Rosenberg et al (2013),^25^ Tanser et al (2011),^26^ Tanser et al (2013),^27^ Akullian et al (2017),^28^ unpublished data from Iwuji et al (2018),^32^ Chimbindi et al (2018),^29^* and unpublished data from Schaefer et al (2017)^41^†	14	20–24	Female	SADC	All	4·36 (3·53–5·39)
Justman et al (2017)^6^ and unpublished data from Schaefer et al (2017)^41^†	5	20–24	Female	SADC	Sensitivity analysis (excluding studies from uMkhanyakude)	1·64 (0·99–2·73)
Dobra et al (2017),^3^ Vandormael et al (2014),^23^ Rosenberg et al (2013),^25^ Tanser et al (2011),^26^ Tanser et al (2013),^27^ Akullian et al (2017),^28^ Chimbindi et al (2018),^29^* and unpublished data from Iwuji et al (2018)^32^	9	20–24	Female	SADC	Sensitivity analysis (uMkhanyakude studies only)	6·86 (5·93–7·93)
Dobra et al (2017),^3^ Justman et al (2017),^6^ Vandormael et al (2014),^23^ Harling et al (2014),^24^ Tanser et al (2011),^26^ Tanser et al (2013),^27^ Akullian et al (2017),^28^ unpublished data from Iwuji et al (2018),^32^ and unpublished data from Schaefer et al (2017)^41^	9	25–29	Female	SADC	All	4·48 (3·19–6·31)
Justman et al (2017)^6^ and unpublished data from Schaefer et al (2017)^41^	2	25–29	Female	SADC	Sensitivity analysis (excluding studies from uMkhanyakude)	1·52 (1·20–1·93)
Dobra et al (2017),^3^ Vandormael et al (2014),^23^ Harling et al (2014),^24^ Tanser et al (2011),^26^ Tanser et al (2013),^27^ Akullian et al (2017),^28^ and unpublished data from Iwuji et al (2018)^32^	7	25–29	Female	SADC	Sensitivity analysis (uMkhanyakude studies only)	5·71 (4·07–8·00)
Dobra et al (2017),^3^ Justman et al (2017),^6^ Vandormael et al (2014),^23^ Tanser et al (2013),^27^ unpublished data from Iwuji et al (2018),^32^ and unpublished data from Schaefer et al (2017)^41^	6	15–19	Male	SADC	All	0·69 (0·52–0·92)
Justman et al (2017)^6^ and unpublished data from Schaefer et al (2017)^41^	2	15–19	Male	SADC	Sensitivity analysis (excluding studies from uMkhanyakude)	0·43 (0·15–1·25)
Dobra et al (2017),^3^ Vandormael et al (2014),^23^ Tanser et al (2013),^27^ and unpublished data from Iwuji et al (2018)^32^	4	15–19	Male	SADC	Sensitivity analysis (uMkhanyakude studies only)	0·84 (0·74–0·97)
Dobra et al (2017),^3^ Justman et al (2017),^6^ Vandormael et al (2014),^23^ Rosenberg et al (2013),^25^ Tanser et al (2013),^27^ Akullian et al (2017),^28^ Baisley et al (2018),^30^* unpublished data from Iwuji et al (2018),^32^ and unpublished data from Schaefer et al (2017)^41^†	13	20–24	Male	SADC	All	1·58 (1·18–2·10)
Justman et al (2017)^6^ and unpublished data from Schaefer et al (2017)^41^†	5	20–24	Male	SADC	Sensitivity analysis (excluding studies from uMkhanyakude)	0·56 (0·29–1·08)
Dobra et al (2017),^3^ Vandormael et al (2014),^23^ Rosenberg et al (2013),^25^ Tanser et al (2013),^27^ Akullian et al (2017),^28^* Baisley et al (2018),^30^ and unpublished data from Iwuji et al (2018)^32^	8	20–24	Male	SADC	Sensitivity analysis (uMkhanyakude studies only)	2·43 (1·87–3·15)
Dobra et al (2017),^3^ Justman et al (2017),^6^ Vandormael et al (2014),^23^ Tanser et al (2013),^27^ Akullian et al (2017),^28^ Baisley et al (2018),^30^† unpublished data from Iwuji et al (2018),^32^ and Feldblum et al (2014)^48^	9	25–29	Male	SADC	All	3·34 (2·58–4·34)
Justman et al (2017)^6^ and unpublished data from Schaefer et al (2017)^41^	2	25–29	Male	SADC	Sensitivity analysis (excluding studies from uMkhanyakude)	1·58 (0·61–4·13)
Dobra et al (2017),^3^ Vandormael et al (2014),^23^ Tanser et al (2013),^27^ Akullian et al (2017),^28^ Baisley et al (2018),^30^† and unpublished data from Iwuji et al (2018)^32^	7	25–29	Male	SADC	Sensitivity analysis (uMkhanyakude studies only)	4·19 (3·48–5·05)
Unpublished data from Ruzagira et al (2011),^14^ Borgdorff et al (2018),^20^ Biraro et al (2013),^36^ unpublished data from Grabowski et al (2017),^37^* Santelli et al (2013),^38^ Santelli et al (2015)^39^*	8	15–19	Female	EAC	All	0·68 (0·45–1·04)
Unpublished data from Ruzagira et al (2011),^14^ Borgdorff et al (2018),^20^ Okiria et al (2014),^35^ Biraro et al (2013),^36^ unpublished data from Grabowski et al (2017),^37^* Santelli et al (2013),^38^ and Santelli et al (2015)^39^*	9	20–24	Female	EAC	All	1·23 (1·01–1·49)
Unpublished data from Ruzagira et al (2011),^14^ Borgdorff et al (2018),^20^ Okiria et al (2014),^35^ Biraro et al (2013),^36^ and unpublished data from Grabowski et al (2017)^37^*	6	25–29	Female	EAC	All	1·07 (0·84–1·36)
Unpublished data from Ruzagira et al (2011),^14^ Biraro et al (2013),^20^ unpublished data from Grabowski et al (2017),^37^* Santelli et al (2013),^38^ and Santelli et al (2015)^39^*	7	15–19	Male	EAC	All	0·21 (0·12–0·37)
Unpublished data from Ruzagira et al (2011),^14^ Borgdorff et al (2018),^20^ Okiria et al (2014),^35^ Biraro et al (2013),^36^ unpublished data from Grabowski et al (2017),^37^* Santelli et al (2013),^38^ and Santelli et al (2015)^39^*	9	20–24	Male	EAC	All	0·72 (0·52–1·02)
Unpublished data from Ruzagira et al (2011),^14^ Borgdorff et al (2018),^20^ Okiria et al (2014),^35^ Biraro et al (2013),^36^ and unpublished data from Grabowski et al (2017)^37^*	6	25–29	Male	EAC	All	1·32 (0·93–1·86)

EAC=East African Community. SADC=Southern African Development Community.

* Two estimates from this study.

† Four estimates from this study.

Overall, the sex differential in HIV risk is greatest among those aged 15–19 years. Meta-regression results offer statistical evidence for a difference in the pooled incidence rate among young women aged 15–19 years in SADC (pooled relative risk 5·94, 95% CI 3·39–10·44), and those aged 20–24 years (2·65, 1·39–5·06), compared with their male counterparts in SADC, and no meaningful difference was found between males and females aged 25–29 years. For the EAC, meta-regression results indicated higher risk among young women aged 15–19 years (pooled relative risk 3·22, 95% CI 1·51–6·87) compared with EAC men of the same age, whereas no sex difference was found in the age groups of 20–24 years and 25–29 years.

## Discussion

Accurate estimates of HIV incidence among targeted groups can be an essential tool in monitoring transmission patterns, determining prevention priorities (in terms of who and where to target efforts), planning interventions, and evaluating progress of programmes. We did a systematic review and meta-analysis to compile recent, direct estimates of HIV incidence among adolescent girls (15–19 years) and young women (20–24 years)—who are increasingly considered to be key populations in HIV prevention strategies—before large investments in targeted prevention began in 2016. Our review has found that estimates based on direct observation of incidence are largely limited to a small number of surveillance sites or relatively small observational and intervention studies with high-risk groups.

From this constrained and heterogeneous body of evidence, some consistent and striking patterns emerge about the risk for young people. First, HIV incidence rates among adolescent females exceed their male peers in all studies and settings. Among the general population in high-burden settings, adolescence is a time in which HIV risk increased rapidly for females, as high as 8% incidence among teenage girls in KwaZulu-Natal, while remaining less than 1% among male peers in almost every study setting.

In the general population studies, female risk continued to increase into their mid-twenties, when it typically peaked in southern African countries. By contrast, in studies of high-risk young women, incidence peaked earlier. Among women sampled from sex workers, bar workers, guest houses, or STI clinics, risk was often higher among teenage girls than older age groups of women. This finding may reflect the vulnerability and the barriers adolescent girls face in accessing testing and treatment services, or negotiating condom use when sex is commercial, transactional, with an older partner, or within marriage. It might also reflect the risk profiles of male partners of high-risk adolescent girls. Alternatively, the lower incidence in older women might be due to saturation in older age groups in high-risk populations (ie, with fewer new people entering these high-risk groups at older ages).

Few studies report trends in HIV incidence since 2005, and the findings are mixed. With previously unpublished age and sex disaggregations of data from ongoing community surveys in Rakai^37^ (Uganda) and Manicaland^41^ (Zimbabwe), and among cohorts of sex workers in Mombasa^45^ (Kenya), there are indications that incidence among adolescent girls and young women is declining. This finding might be due to the indirect effects of ART coverage in the community, as well as adoption of safer sexual behaviours including condom use, or epidemic stage or demographic change.^70^, ^71^ However, rates among adolescent girls and young women remain high (above elimination targets), and persistently higher than male peers. Also, in the highest incidence settings of South Africa, as in KwaZulu-Natal and fishing communities in Uganda, there was no evidence of a decline by 2015. This result suggests that—among other factors—availability of ART had not benefited the highest risk young women; for example, their sexual partners might not be aware of their HIV status or linking into care and treatment to achieve viral suppression, as recent results of the TasP trial indicate.^72^ Indeed, ART coverage has been lower among HIV-positive men in their twenties and thirties (commonly the age group for male sexual partners of adolescent girls and young women), relative to women and older adults, and this gender discrepancy has been most pronounced in sub-Saharan Africa.^28^ Furthermore, high HIV incidence rates observed among young adult men might reflect recent infection and high infectiousness among male sexual partners of adolescent girls and young women, before they become aware of their status and link into care.

The national estimates from South Africa in 2005 and 2012 suggest a decline in HIV incidence; however, the use of different assay methodologies precludes their comparability. The earlier survey used the BED assay, which is susceptible to overestimation of incidence rates (upward bias) in Africa, without the application of context-specific correction factors.^73^ More recent national surveys across multiple countries have used limiting antigen avidity enzyme immunoassays using WHO-recommended algorithms for recent infection,^68^ and might be used to track trends in incidence over time. For example, recent Swaziland PHIA results, as shared in a 2017 conference,^74^ were compared with estimates from the SHIMS 2010–11 cohort study^5^ to conclude that HIV incidence has declined in Swaziland. However, the comparability between estimates derived from repeat testing within cohorts and from assays is unknown, and studies using both methods are needed for validation. There are also questions about the interpretation of estimates from assays, given the policy shift towards immediate treatment meaning that even recently infected individuals will increasingly be on treatment, as well as the need for viral load data, and the questionable performance of assays (eg, wide ranges in false recency rates and mean duration of recent infection, and high misclassification rates) in different epidemiological contexts and HIV subtypes.^73^, ^75^ Furthermore, the very large samples required for estimation—and the relatively small number of recent infections identified in the PHIAs—can limit meaningful disaggregation of estimates by age, sex, and subnational area, to effectively identify the priority places and populations for prevention.

The studies we identified are highly variable in terms of study design, size, population, and timing. Where possible from available data, we pooled estimates for general population studies and conclude that this exercise is influenced by geographical skew (preponderance of studies from the same surveillance sites) and hampered by heterogeneity. It also minimises the geographical, demographic, and epidemiological differences that must be understood to orient programming efforts. Going forward, tracking progress of such efforts will rely on greater comparability between studies, especially over time (more so than place), given the few trend analyses available to date.

It is possible that the studies reviewed here represent the highest estimates among young people—eg, through publication bias for which authors are more likely to report age breakdowns or in which journals are more likely to publish results with high numbers of seroconversions. When we requested disaggregations by age and sex, from 18 authors who had published HIV incidence but not specifically for young women, the number of seroconversions in the unpublished reports were generally low and CIs often wide—possibly explaining why some of these estimates were not published previously. We received no data from eight of those studies and their omission might have distorted the final sample.

It is also possible that some published estimates appear higher than they might be for the youngest populations, because of methods that impute the timing of seroconversion from the midpoint (between last negative and first positive HIV test)—the method used by most analyses in the review. Among young people, the timing of infection is likely to be recent (ie, more recent than the midpoint), and use of the midpoint might inflate estimates at younger ages, especially if intervals between testing are long. Another probable influence is the need for parental consent among participants younger than 18 years; parental consent might impede participation of young people most likely to be home with parents (and less likely to be sexually active). Also, participation might be biased towards sexually active adolescents in studies where emancipated minors (eg, married or pregnant youth) have autonomy to consent. Finally, the most recent estimates will be overestimated if there is a delay in repeat testing among HIV-negative individuals, and the most current estimates might be corrected as more data become available.

There are also methodological reasons why the rates might be underestimated. Most published estimates are drawn from community surveillance areas, most of which are rural settings where incidence might be lower than in other parts of the country. Participation levels in sero-survey rounds were not always reported, and can be low because of research fatigue (at individual and population levels).^76^ Those who participate and consent to repeated HIV testing might be at lower risk than those who refuse or avoid participation and those who move in and out of the surveillance area or are unavailable (with mobility being associated with HIV risk in South Africa). Over time, as people learn their HIV status sooner after infection, those already diagnosed with HIV might have less incentive to participate in a sero-survey, which could skew the incidence estimates downward. Research from KwaZulu-Natal suggests participation bias can reduce the accuracy with which seroconversions can be dated, undermining validity, but does not introduce a systematic bias.^76^

The method of estimating HIV incidence might also underestimate rates—eg, with young people being less able to meet the need to have tested twice (they often miss a survey round because they are slightly too young to participate). Repeated testing is more likely to occur in sites with frequent opportunities to test and where participation is high. Where a substantial proportion of young people have tested only once, because of age restrictions, it is desirable to impute a negative status at a suitable age to include their data. If young prevalent positive cases are discarded, the resulting estimates will be lower, since few young seroconverters will have had an earlier observation while HIV negative.

The results of this review will also be influenced by limitations of our search terms, strategy, and execution. For example, by not systematically searching conference abstracts or non-English language articles, we might have missed eligible estimates. Although accuracy and completeness of the estimates cannot be certain, and the results cannot be generalised or extrapolated beyond the study settings, this review shows that adolescent girls' risk cannot be minimised. The pattern of rapidly increasing risk, from an earlier age in comparison with male peers, is consistent across settings and in the pooled analyses. The indirect benefits of ART are slow to yield reductions in the highest-prevalence settings, particularly where HIV testing and treatment among young adult men remain low. The sparse trend data available in this review indicate that absolute levels of incidence might well be declining, in various settings and populations, before investments targeting adolescent girls and young women such as DREAMS, but rates remain unacceptably high and the gender disparity during adolescence and early adulthood persists. Programmes such as DREAMS can tackle the underlying gender inequalities that drive disproportionately high risk among girls. Furthermore, where HIV incidence estimates are available in districts before DREAMS implementation—as is the case for several studies included in this review—such data are a valuable source with which to verify whether DREAMS initiates or accelerates declines in risk and achieves its important aims of reducing HIV infections among adolescent girls and young women in sub-Saharan Africa.
