# Supplementary material for: Recent levels and trends in HIV incidence rates among adolescent girls and young women in ten high-prevalence African countries: a systematic review and meta-analysis
Source: Lancet Glob Health. 2019 Oct 10;7(11):e1521–40. doi: 10.1016/S2214-109X(19)30410-3 (PMC7025003; doi:10.1016/S2214-109X(19)30410-3)
Supplement: Supplementary appendix [file mmc1.pdf]

THE LANCET
Global Health

## Supplementary appendix

This appendix formed part of the original submission and has been peer reviewed. We post it as supplied by the authors.

Supplement to: Birdthistle I, Tanton C, Tomita A, et al. Recent levels and trends in HIV incidence rates among adolescent girls and young women in ten high-prevalence African countries: a systematic review and meta-analysis. *Lancet Glob Health* 2019; **7:** e1521–40.

# Supplementary Material

## Contents

*Table S1. Studies that met the inclusion criteria but do not report HIV incidence disaggregated by sex and/or young age groups* .................. 2

*Table S2. General population-based study characteristics and data extracted for review and pooled analyses* ................................................. 5

*Table S1. Studies that met the inclusion criteria but do not report HIV incidence disaggregated by sex and/or young age groups*

| ***Country*** | ***Author, year of publication*** | ***Period of data collection*** | ***Location, region, setting*** | ***Population*** | ***Study Design (measure of HIV incidence)*** |
|---|---|---|---|---|---|
| ***Kenya*** | Otieno, et al. (2015) [1] | 2007-2009 | Kisumu (KiCOS) | Adults aged 18-34 years | Cohort (direct) |
| | Gumbe, et al. (2015) [2] | 2007-2009 | Kisumu (KiCOS) | Adults aged 16-34 years | Cohort (direct) |
| ***Malawi*** | Keating, et al. (2012) [3] | | Lilongwe (Bwaila hospital) | Pregnant women | Retrospective cohort (direct) |
| ***Mozambique*** | Perez-Hoyos, et al. (2011) [4] | 1999-2008 | Manhica (Antenatal Clinic) | Women aged 15-45 years | Incidence estimated from prevalence data |
| | Mocumbi, et al (2017) [10] | 2007-2009 | Urban Mavalane & rural Manhica | Sexually active, HIV negative woman aged 18+ yrs | Cohort, clinical (direct) |
| ***Uganda*** | Guwatudde, et al. (2009) [5] | 2006 | Kayunga District | Adults aged 15-49 years | Cohort (direct) |
| | Kiwanuka, et al. (2014) [6] | 2011-2013 | 8 fishing communities (Community-based) | Adults aged 18-49 | Cohort (direct) |
| | Kouyoumdijian, et al. (2013) [7] | 2000-2009 | Rakai | Adults aged 15-49 | Cohort (direct) |
| ***South Africa*** | Huerga, et al. (2017) [11] | 2013 | KwaZulu-Natal (Mbongolwane & Eshowe) | Adults aged 15-59 | Recent HIV incidence (Lag-Avidity testing) |
| | Nel, et al. (2012) [8] | 2007-2010 | KwaZulu-Natal (Ladysmith, Edendale & Pinetown) | Women aged 18-35 years | Cohort (direct) |

| | | | | |
|---|---|---|---|---|
| Abdool Karim, et al (2011) [12] | 2005-2008 | Malawi (Blantyre & Lilongwe), South Africa (Durban & Hlabisa), Zambia (Lusaka), Zimbabwe (Harare & Chitungwiza) | Women aged 17-56 years and HIV-negative, non-pregnant, sexually active (vaginal intercourse >=1 in past 3 months) | Cohort (direct) analysis from trial data |
| Ramjee, et al. (2012) [9] | 2004-2009 | Durban | Women | Cohort (direct) analysis from two trials |

**References:**


1. Otieno FO, Ndivo R, Oswago S, Pals S, Chen R, Thomas T, et al. Correlates of prevalent sexually transmitted infections among participants screened for an HIV incidence cohort study in Kisumu, Kenya. Int J STD AIDS. 2015; 26(4): 225-37.
2. Gumbe A, McLellan-Lemal E, Gust DA, Pals SL, Gray KM, Ndivo R, et al. Correlates of prevalent HIV infection among adults and adolescents in the Kisumu incidence cohort study, Kisumu, Kenya. International Journal of STD and AIDS. 2015; 26(13): 929-40.
3. Keating MA, Hamela G, Miller WC, Moses A, Hoffman IF, Hosseinipour MC. High HIV incidence and sexual behavior change among pregnant women in Lilongwe, Malawi: implications for the risk of HIV acquisition. PLoS ONE [Electronic Resource]. 2012; 7(6): e39109.
4. Perez-Hoyos S, Naniche D, Macete E, Aponte J, Sacarlal J, Sigauque B, et al. Stabilization of HIV incidence in women of reproductive age in southern Mozambique. HIV Med. 2011; 12(8): 500-5 6p.
5. Guwatudde D, Wabwire-Mangen F, Eller LA, Eller M, McCutchan F, Kibuuka H, et al. Relatively low HIV infection rates in rural Uganda, but with high potential for a rise: A cohort study in Kayunga District, Uganda. PLoS ONE. 2009; 4 (1):e4145).
6. Kiwanuka N, Mpendo J, Nalutaaya A, Wambuzi M, Nanvubya A, Kitandwe PK, et al. An assessment of fishing communities around Lake Victoria, Uganda, as potential populations for future HIV vaccine efficacy studies: an observational cohort study. BMC Public Health. 2014; 14(1): 986- 1p.
7. Kouyoumdjian FG, Calzavara LM, Bondy SJ, O'Campo P, Serwadda D, Nalugoda F, et al. Intimate partner violence is associated with incident HIV infection in women in Uganda. AIDS. 2013; 27(8): 1331-8.
8. Nel A, Mabude Z, Smit J, Kotze P, Arbuckle D, Wu J, et al. HIV incidence remains high in KwaZulu-Natal, South Africa: evidence from three districts. PLoS ONE [Electronic Resource]. 2012; 7(4): e35278.
9. Ramjee G, Wand H. Population-level impact of hormonal contraception on incidence of HIV infection and pregnancy in women in Durban, South Africa. Bull World Health Organ. 2012; 90(10): 748-55.
10. Mocumbi S, Gafos M, Munguambe K, Goodall R. McCormack S. High HIV prevalence and incidence among women in Southern Mozambique: Evidence from the MDP microbicide feasibility study. PLoS One. 2017; 12(3):e0173243.
11. Huerga H, Shiferie F, Grebe E, Giuliani R, Farhat JB, Van-Cutsem G, Cohen K. A comparison of self-report and antiretroviral detection to inform estimates of antiretroviral therapy coverage, viral load suppression and HIV incidence in Kwazulu-Natal, South Africa. BMC Infectious Diseases. 2017; 17(1): 653.
12. Abdool Karim Q, Kharsany AB, Frohlich, JA, Werner L, Mashego M, Mlotshwa M, Madlala BT, Ntombela F, Abdool Karim SS. Stabilizing HIV prevalence masks high HIV incidence rates amongst rural and urban women in KwaZulu-Natal, South Africa. Intl J of Epidemiology. 2011; 40(4): 922-30.

*Table S2. General population-based study characteristics and data extracted for review and pooled analyses*

| ID | Country | Region | Study | Year | Sex | Age Group | Incidence | Lower Bound | Upper Bound | Quality appraisal for non-assay-based studies |
|---|---|---|---|---|---|---|---|---|---|---|
| 1 | Malawi | SADC | National | 2015–16 | Female subgroup | 15–24 | 0.40 | 0.04 | 0.77 | |
| 2 | Malawi | SADC | National | 2015–16 | Male subgroup | 15–24 | 0.05 | 0.00 | 0.19 | |
| 3 | Malawi | SADC | National | 2015–16 | Female subgroup | 25–34 | 0.87 | 0.11 | 1.63 | |
| 4 | Malawi | SADC | National | 2015–16 | Male subgroup | 25–34 | 0.40 | 0.00 | 0.91 | |
| 5 | South Africa | SADC | KZN, uMkhanyakude district | 2004–12 | Female subgroup | 15–19 | 4.91 | 4.48 | 5.39 | 100 |
| 6 | South Africa | SADC | KZN, uMkhanyakude district | 2004–12 | Male subgroup | 15–19 | 0.90 | 0.71 | 1.12 | 100 |
| 7 | South Africa | SADC | KZN, uMkhanyakude district | 2004–12 | Female subgroup | 20–24 | 7.80 | 7.19 | 8.46 | 100 |
| 8 | South Africa | SADC | KZN, uMkhanyakude district | 2004–12 | Male subgroup | 20–24 | 3.28 | 2.85 | 3.78 | 100 |
| 9 | South Africa | SADC | KZN, uMkhanyakude district | 2004–12 | Female subgroup | 25–29 | 6.50 | 5.66 | 7.45 | 100 |
| 10 | South Africa | SADC | KZN, uMkhanyakude district | 2004–12 | Male subgroup | 25–29 | 4.66 | 3.82 | 5.68 | 100 |
| 11 | South Africa | SADC | KZN, uMkhanyakude district | 2003–12 | Female subgroup | 15–29 | | | | 90 |
| 12 | South Africa | SADC | KZN, uMkhanyakude district | 2003–12 | Female subgroup | 15–19 | 7.79 | 6.59 | 9.22 | 90 |
| 13 | South Africa | SADC | KZN, uMkhanyakude district | 2003–12 | Female subgroup | 20–24 | 8.63 | 7.63 | 9.77 | 90 |
| 14 | South Africa | SADC | KZN, uMkhanyakude district | 2003–12 | Female subgroup | 25–29 | 5.63 | 4.46 | 7.11 | 90 |
| 15 | South Africa | SADC | KZN, uMkhanyakude district | 2006–11 | Female subgroup | 15–24 | 4.37 | 3.79 | 5.04 | 80 |
| 16 | South Africa | SADC | KZN, uMkhanyakude district | 2006–11 | Male subgroup | 15–24 | 1.38 | 1.07 | 1.79 | 80 |
| 17 | South Africa | SADC | KZN, uMkhanyakude district | 2004–09 | Female subgroup | 15–19 | 5.10 | 4.58 | 5.67 | 100 |

| | | | | | | | | | | |
|---|---|---|---|---|---|---|---|---|---|---|
| 18 | South Africa | SADC | KZN, uMkhanyakude district | 2004–09 | Female subgroup | 20–24 | 7.47 | 6.33 | 8.76 | 100 |
| 19 | South Africa | SADC | KZN, uMkhanyakude district | 2004–09 | Female subgroup | 25–29 | 5.18 | 3.88 | 6.77 | 100 |
| 20 | South Africa | SADC | KZN, uMkhanyakude district | 2004–11 | Female subgroup | 15–19 | 4.43 | 3.96 | 4.95 | 90 |
| 21 | South Africa | SADC | KZN, uMkhanyakude district | 2004–11 | Male subgroup | 15–19 | 0.74 | 0.56 | 0.99 | 90 |
| 22 | South Africa | SADC | KZN, uMkhanyakude district | 2004–11 | Female subgroup | 20–24 | 6.49 | 5.86 | 7.18 | 90 |
| 23 | South Africa | SADC | KZN, uMkhanyakude district | 2004–11 | Male subgroup | 20–24 | 2.53 | 2.10 | 3.05 | 90 |
| 24 | South Africa | SADC | KZN, uMkhanyakude district | 2004–11 | Female subgroup | 25–29 | 5.51 | 4.64 | 6.54 | 90 |
| 25 | South Africa | SADC | KZN, uMkhanyakude district | 2004–11 | Male subgroup | 25–29 | 4.43 | 3.50 | 5.61 | 90 |
| 26 | South Africa | SADC | KZN, uMkhanyakude district | 2004–11 | Female subgroup | 15–19 | 5.10 | 4.61 | 5.6 | 90 |
| 27 | South Africa | SADC | KZN, uMkhanyakude district | 2004–11 | Male subgroup | 15–19 | 0.91 | 0.7 | 1.12 | 90 |
| 28 | South Africa | SADC | KZN, uMkhanyakude district | 2004–11 | Female subgroup | 20–24 | 9.11 | 8.4 | 9.82 | 90 |
| 29 | South Africa | SADC | KZN, uMkhanyakude district | 2004–11 | Male subgroup | 20–24 | 3.69 | 3.19 | 4.19 | 90 |
| 30 | South Africa | SADC | KZN, uMkhanyakude district | 2004–11 | Female subgroup | 25–29 | 7.03 | 6.07 | 7.99 | 90 |
| 31 | South Africa | SADC | KZN, uMkhanyakude district | 2004–11 | Male subgroup | 25–29 | 5.79 | 4.79 | 6.80 | 90 |
| 32 | South Africa | SADC | KZN, uMkhanyakude district | 2003–07 | Female subgroup | 15–19 | 3.9 | 2.9 | 5.3 | 90 |
| 33 | South Africa | SADC | KZN, uMkhanyakude district | 2003–07 | Male subgroup | 15–19 | 1.0 | 0.5 | 1.8 | 90 |
| 34 | South Africa | SADC | KZN, uMkhanyakude district | 2003–07 | Female subgroup | 20–24 | 5.6 | 4.0 | 8.0 | 90 |
| 35 | South Africa | SADC | KZN, uMkhanyakude district | 2003–07 | Male subgroup | 20–24 | 2.8 | 1.6 | 4.8 | 90 |
| 36 | South Africa | SADC | KZN, uMkhanyakude district | 2003–07 | Female subgroup | 25–29 | 8.0 | 4.9 | 13.0 | 90 |

| | | | | | | | | | | |
|---|---|---|---|---|---|---|---|---|---|---|
| 37 | South Africa | SADC | KZN, uMkhanyakude district | 2003–07 | Male subgroup | 25–29 | 8.7 | 4.8 | 15.8 | 90 |
| 38 | South Africa | SADC | KZN, uMkhanyakude district | 2004–15 | Female subgroup | 15–24 | 5.6 | 5.3 | 5.9 | 90 |
| 39 | South Africa | SADC | KZN, uMkhanyakude district | 2004–15 | Male subgroup | 15–24 | 1.7 | 1.5 | 1.9 | 90 |
| 40 | South Africa | SADC | KZN, uMkhanyakude district | 2004–15 | Female subgroup | 25–49 | 3.1 | 2.9 | 3.4 | 90 |
| 41 | South Africa | SADC | KZN, uMkhanyakude district | 2004–15 | Male subgroup | 25–49 | 3.6 | 3.1 | 4.2 | 90 |
| 42 | South Africa | SADC | KZN, uMkhanyakude district | 2006–10 | Female subgroup | 15–19 | 4.71 | 4.10 | 5.41 | 90 |
| 43 | South Africa | SADC | KZN, uMkhanyakude district | 2011–15 | Female subgroup | 15–19 | 4.54 | 3.89 | 5.30 | 90 |
| 44 | South Africa | SADC | KZN, uMkhanyakude district | 2006–10 | Female subgroup | 20–24 | 7.62 | 6.71 | 8.65 | 90 |
| 45 | South Africa | SADC | KZN, uMkhanyakude district | 2011–15 | Female subgroup | 20–24 | 7.45 | 6.51 | 8.51 | 90 |
| 46 | South Africa | SADC | KZN, uMkhanyakude district | 2006–10 | Male subgroup | 20–24 | 3.08 | 2.49 | 3.82 | 90 |
| 47 | South Africa | SADC | KZN, uMkhanyakude district | 2011–15 | Male subgroup | 20–24 | 2.58 | 2.00 | 3.32 | 90 |
| 48 | South Africa | SADC | KZN, uMkhanyakude district | 2006–10 | Male subgroup | 25–29 | 4.43 | 3.34 | 5.87 | 90 |
| 49 | South Africa | SADC | KZN, uMkhanyakude district | 2011–15 | Male subgroup | 25–29 | 4.04 | 3.07 | 5.31 | 90 |
| 50 | South Africa | SADC | KZN, uMkhanyakude district | 2012–15 | Female subgroup | 16–19 | 5.54 | 4.64 | 6.45 | 70 |
| 51 | South Africa | SADC | KZN, uMkhanyakude district | 2012–15 | Male subgroup | 16–19 | 0.61 | 0.28 | 0.94 | 70 |
| 52 | South Africa | SADC | KZN, uMkhanyakude district | 2012–15 | Female subgroup | 20–24 | 6.93 | 5.75 | 8.11 | 70 |
| 53 | South Africa | SADC | KZN, uMkhanyakude district | 2012–15 | Male subgroup | 20–24 | 2.02 | 1.19 | 2.84 | 70 |
| 54 | South Africa | SADC | KZN, uMkhanyakude district | 2012–15 | Female subgroup | 25–29 | 6.34 | 4.77 | 7.90 | 70 |
| 55 | South Africa | SADC | KZN, uMkhanyakude district | 2012–15 | Male subgroup | 25–29 | 1.06 | 0.21 | 1.90 | 70 |

| | | | | | | | | | | |
|---|---|---|---|---|---|---|---|---|---|---|
| 56 | South Africa | SADC | National | 2005–05 | Female subgroup | 15–24 | 6.5 | 2.3 | 10.7 | 100 |
| 57 | South Africa | SADC | National | 2005–05 | Male subgroup | 15–24 | 0.8 | 0 | 3.4 | 100 |
| 58 | South Africa | SADC | National | 2012–12 | Female subgroup | 15–24 | 2.54 | 2.04 | 3.04 | 100 |
| 59 | South Africa | SADC | National | 2012–12 | Male subgroup | 15–24 | 0.55 | 0.45 | 0.65 | 100 |
| 60 | South Africa | SADC | National | 2012–12 | Female subgroup | 25+ | 1.62 | 1.30 | 1.94 | 100 |
| 61 | South Africa | SADC | National | 2012–12 | Male subgroup | 25+ | 1.29 | 0.91 | 1.67 | 100 |
| 62 | Swaziland | SADC | National | 2010–11 | Female subgroup | 18–19 | 3.8 | 2.6 | 5.6 | 100 |
| 63 | Swaziland | SADC | National | 2010–11 | Male subgroup | 18–19 | 0.8 | 0.4 | 1.9 | 100 |
| 64 | Swaziland | SADC | National | 2010–11 | Female subgroup | 20–24 | 4.3 | 3.3 | 5.6 | 100 |
| 65 | Swaziland | SADC | National | 2010–11 | Male subgroup | 20–24 | 1.6 | 1.1 | 2.5 | 100 |
| 66 | Swaziland | SADC | National | 2010–11 | Female subgroup | 25–29 | 2.0 | 1.2 | 3.2 | 100 |
| 67 | Swaziland | SADC | National | 2010–11 | Male subgroup | 25–29 | 2.6 | 1.7 | 4.0 | 100 |
| 68 | Tanzania | EAC | Mbeya | 2005–06 | Female subgroup | 20–24 | 2.60 | 1.51 | 4.47 | 70 |
| 69 | Tanzania | EAC | Mbeya | 2005–06 | Male subgroup | 20–24 | 1.36 | 0.57 | 3.27 | 70 |
| 70 | Tanzania | EAC | Mbeya | 2005–06 | Female subgroup | 25–29 | 0.26 | 0.04 | 1.84 | 70 |
| 71 | Tanzania | EAC | Mbeya | 2005–06 | Male subgroup | 25–29 | 0.72 | 0.18 | 2.88 | 70 |
| 72 | Uganda | EAC | Masaka | 1990–2007 | Female subgroup | 13–19 | 0.57 | 0.44 | 0.74 | 90 |
| 73 | Uganda | EAC | Masaka | 1990–2007 | Male subgroup | 13–19 | 0.09 | 0.04 | 0.16 | 90 |
| 74 | Uganda | EAC | Masaka | 1990–2007 | Female subgroup | 20–24 | 1.19 | 0.91 | 1.53 | 90 |
| 75 | Uganda | EAC | Masaka | 1990–2007 | Male subgroup | 20–24 | 0.79 | 0.55 | 1.09 | 90 |
| 76 | Uganda | EAC | Masaka | 1990–2007 | Female subgroup | 25–29 | 0.95 | 0.68 | 1.3 | 90 |
| 77 | Uganda | EAC | Masaka | 1990–2007 | Male subgroup | 25–29 | 1.35 | 0.99 | 1.8 | 90 |

| | | | | | | | | | | |
|---|---|---|---|---|---|---|---|---|---|---|
| 78 | Uganda | EAC | Masaka | 2005–11 | Female subgroup | 15–19 | 1.03 | 0.64 | 1.57 | 90 |
| 79 | Uganda | EAC | Masaka | 2011–16 | Female subgroup | 15–19 | 0.59 | 0.31 | 1.01 | 90 |
| 80 | Uganda | EAC | Masaka | 2005–11 | Male subgroup | 15–19 | 0.30 | 0.12 | 0.6 | 90 |
| 81 | Uganda | EAC | Masaka | 2011–16 | Male subgroup | 15–19 | 0.16 | 0.05 | 0.38 | 90 |
| 82 | Uganda | EAC | Masaka | 2005–11 | Female subgroup | 20–24 | 1.47 | 1.12 | 1.9 | 90 |
| 83 | Uganda | EAC | Masaka | 2011–16 | Female subgroup | 20–24 | 1.53 | 1.13 | 2.01 | 90 |
| 84 | Uganda | EAC | Masaka | 2005–11 | Male subgroup | 20–24 | 0.9 | 0.61 | 1.28 | 90 |
| 85 | Uganda | EAC | Masaka | 2011–16 | Male subgroup | 20–24 | 0.4 | 0.21 | 0.68 | 90 |
| 86 | Uganda | EAC | Masaka | 2005–11 | Female subgroup | 25–29 | 1.47 | 1.15 | 1.84 | 90 |
| 87 | Uganda | EAC | Masaka | 2011–16 | Female subgroup | 25–29 | 1.12 | 0.81 | 1.5 | 90 |
| 88 | Uganda | EAC | Masaka | 2005–11 | Male subgroup | 25–29 | 1.73 | 1.31 | 2.23 | 90 |
| 89 | Uganda | EAC | Masaka | 2011–16 | Male subgroup | 25–29 | 1.26 | 0.89 | 1.72 | 90 |
| 90 | Uganda | EAC | Kumi district | 2006–08 | Female subgroup | 13–24 | 0.38 | 0.19 | 0.66 | 70 |
| 91 | Uganda | EAC | Kumi district | 2006–08 | Male subgroup | 13–24 | 0.26 | 0.07 | 0.66 | 70 |
| 92 | Uganda | EAC | Kumi district | 2006–08 | Female subgroup | 25–34 | 0.68 | 0.44 | 1.01 | 70 |
| 93 | Uganda | EAC | Kumi district | 2006–08 | Male subgroup | 25–34 | 0.28 | 0.09 | 0.65 | 70 |
| 94 | Uganda | EAC | Rakai | 1998–2008 | Female subgroup | 15–19 | 1.49 | 1.06 | 2.04 | 90 |
| 95 | Uganda | EAC | Rakai | 1998–2008 | Male subgroup | 15–19 | 0.36 | 0.14 | 0.73 | 90 |
| 96 | Uganda | EAC | Rakai | 1998–2008 | Female subgroup | 20–24 | 1.38 | 1.13 | 1.66 | 90 |
| 97 | Uganda | EAC | Rakai | 1998–2008 | Male subgroup | 20–24 | 1.02 | 0.76 | 1.35 | 90 |
| 98 | Uganda | EAC | Rakai | 2006–09 | Female subgroup | 15–19 | 0.63 | 0.20 | 1.47 | 90 |
| 99 | Uganda | EAC | Rakai | 2006–09 | Male subgroup | 15–19 | 0.22 | 0.03 | 0.79 | 90 |

| | | | | | | | | | | |
|---|---|---|---|---|---|---|---|---|---|---|
| 100 | Uganda | EAC | Rakai | 2006–09 | Female subgroup | 20–24 | 1.31 | 0.82 | 1.98 | 90 |
| 101 | Uganda | EAC | Rakai | 2006–09 | Male subgroup | 20–24 | 0.79 | 0.38 | 1.45 | 90 |
| 102 | Uganda | EAC | Rakai | 2008–11 | Female subgroup | 15–19 | 0.23 | 0.03 | 0.83 | 90 |
| 103 | Uganda | EAC | Rakai | 2008–11 | Male subgroup | 15–19 | 0.19 | 0.02 | 0.69 | 90 |
| 104 | Uganda | EAC | Rakai | 2008–11 | Female subgroup | 20–24 | 1.55 | 0.95 | 2.39 | 90 |
| 105 | Uganda | EAC | Rakai | 2008–11 | Male subgroup | 20–24 | 1.23 | 0.69 | 2.03 | 90 |
| 106 | Uganda | EAC | Masaka | 2004–07 | Female subgroup | 15–19 | 0 | | | 70 |
| 107 | Uganda | EAC | Masaka | 2004–07 | Male subgroup | 15–19 | 0 | | | 70 |
| 108 | Uganda | EAC | Masaka | 2004–07 | Female subgroup | 20–24 | 0 | | | 70 |
| 109 | Uganda | EAC | Masaka | 2004–07 | Male subgroup | 20–24 | 2.5 | 0.8 | 7.7 | 70 |
| 110 | Uganda | EAC | Masaka | 2004–07 | Female subgroup | 25–29 | 2.0 | 0.8 | 4.8 | 70 |
| 111 | Uganda | EAC | Masaka | 2004–07 | Male subgroup | 25–29 | 2.2 | 0.7 | 6.8 | 70 |
| 112 | Zambia | SADC | National | 2015–16 | Female subgroup | 15–24 | 1.07 | 0.52 | 1.62 | |
| 113 | Zambia | SADC | National | 2015–16 | Male subgroup | 15–24 | 0.08 | 0.00 | 0.25 | |
| 114 | Zambia | SADC | National | 2015–16 | Female subgroup | 25–34 | 1.16 | 0.46 | 1.86 | |
| 115 | Zambia | SADC | National | 2015–16 | Male subgroup | 25–34 | 0.25 | 0.00 | 0.63 | |
| 116 | Kenya | EAC | Gem, Siaya County | 2011–16 | Female subgroup | 15–19 | 0.43 | 2.96 | 5.91 | 90 |
| 117 | Kenya | EAC | Gem, Siaya County | 2011–16 | Female subgroup | 20–24 | 1.12 | 0.8 | 1.52 | 90 |
| 118 | Kenya | EAC | Gem, Siaya County | 2011–16 | Male subgroup | 15–24 | 0.32 | 0.19 | 0.51 | 90 |
| 119 | Kenya | EAC | Gem, Siaya County | 2011–16 | Female subgroup | 25–34 | 0.96 | 0.73 | 1.25 | 90 |
| 120 | Kenya | EAC | Gem, Siaya County | 2011–16 | Male subgroup | 25–34 | 1.07 | 0.71 | 1.57 | 90 |

| | | | | | | | | | | |
|---|---|---|---|---|---|---|---|---|---|---|
| 121 | Kenya | EAC | Ndhiwaza sub-county | 2012 | Female subgroup | 15–24 | 2.07 | 0.85 | 3.29 | |
| 122 | Kenya | EAC | Ndhiwaza sub-county | 2012 | Female subgroup | 15–24 | 2.5 | 1.1 | 4.5 | |
| 123 | Kenya | EAC | Ndhiwaza sub-county | 2012 | Male subgroup | 15–24 | 0.25 | 0.00 | 0.73 | |
| 124 | Kenya | EAC | Ndhiwaza sub-county | 2012 | Male subgroup | 15–24 | 0.3 | 0.00 | 2.7 | |
| 125 | Kenya | EAC | Ndhiwaza sub-county | 2012 | Female subgroup | 25–34 | 1.9 | 0.00 | 3.9 | |
| 126 | Kenya | EAC | Ndhiwaza sub-county | 2012 | Female subgroup | 25–34 | 2.4 | 0.2 | 5.1 | |
| 127 | Kenya | EAC | Ndhiwaza sub-county | 2012 | Male subgroup | 25–34 | 2.5 | 0.2 | 4.6 | |
| 128 | Kenya | EAC | Ndhiwaza sub-county | 2012 | Male subgroup | 25–34 | 0.96 | 0.00 | 2.79 | |
| 129 | Zimbabwe | SADC | Manicaland | 2003–13 | Female subgroup | 15–19 | 0.99 | 0.71 | 1.38 | 90 |
| 130 | Zimbabwe | SADC | Manicaland | 2004–08 | Female subgroup | 15–24 | 1.94 | 1.50 | 2.51 | 90 |
| 131 | Zimbabwe | SADC | Manicaland | 2006–11 | Female subgroup | 15–24 | 0.72 | 0.46 | 1.14 | 90 |
| 132 | Zimbabwe | SADC | Manicaland | 2009–13 | Female subgroup | 15–24 | 1.12 | 0.72 | 1.76 | 90 |
| 133 | Zimbabwe | SADC | Manicaland | 2003–13 | Female subgroup | 20–24 | 1.62 | 1.26 | 2.08 | 90 |
| 134 | Zimbabwe | SADC | Manicaland | 2003–13 | Male subgroup | 15–19 | 0.26 | 0.15 | 0.46 | 90 |
| 135 | Zimbabwe | SADC | Manicaland | 2004–08 | Male subgroup | 15–24 | 0.93 | 0.64 | 1.36 | 90 |
| 136 | Zimbabwe | SADC | Manicaland | 2006–11 | Male subgroup | 15–24 | 0.15 | 0.06 | 0.39 | 90 |
| 137 | Zimbabwe | SADC | Manicaland | 2009–13 | Male subgroup | 15–24 | 0.19 | 0.06 | 0.60 | 90 |
| 138 | Zimbabwe | SADC | Manicaland | 2003–13 | Male subgroup | 20–24 | 0.83 | 0.55 | 1.26 | 90 |
| 139 | Zimbabwe | SADC | Manicaland | 2003–13 | Female subgroup | 25–29 | 1.45 | 1.12 | 1.88 | 90 |
| 140 | Zimbabwe | SADC | Manicaland | 2003–13 | Male subgroup | 25–29 | 1.47 | 1.04 | 2.06 | 90 |
| 141 | Zimbabwe | SADC | National | 2015–16 | Female subgroup | 15–24 | 0.53 | 0.13 | 0.93 | |
| 142 | Zimbabwe | SADC | National | 2015–16 | Male subgroup | 15–24 | 0.14 | 0.00 | 0.37 | |

| | | | | | | | | | |
|---|---|---|---|---|---|---|---|---|---|
| 143 | Zimbabwe | SADC | National | 2015–16 | Female subgroup | 25–34 | 1.11 | 0.41 | 1.80 |
| 144 | Zimbabwe | SADC | National | 2015–16 | Male subgroup | 25–34 | 0.48 | 0.00 | 1.05 |
